# Carrier Diffusion Links Single Crystal Quality and Photoluminescence in Halide Perovskite Radiation Detectors

**DOI:** 10.1002/adma.202512302

**Published:** 2025-10-16

**Authors:** Zimu Wei, Khasim Saheb Bayikadi, Capucine Mamak, Milos Dubajic, Chieh‐Szu Huang, Linfeng Pan, Mercouri G. Kanatzidis, Samuel D. Stranks

**Affiliations:** ^1^ Department of Chemical Engineering and Biotechnology University of Cambridge Cambridge CB3 0AS UK; ^2^ Department of Chemistry Northwestern University Evanston IL 60208 USA

**Keywords:** carrier diffusion, defects, perovskites, photoluminescence, radiation detectors, simulations, single crystals

## Abstract

Halide perovskites have emerged as promising materials for next‐generation radiation detectors, echoing their transformative impact on photovoltaics. Due to the long penetration depths of X‐rays and γ‐rays, thick single crystals are required to sufficiently attenuate the radiation, making bulk crystal quality critical for device performance. Photoluminescence properties, particularly long lifetimes and redshifted emission peaks, are commonly used as proxies for identifying high‐quality CsPbBr_3_ crystals for high‐performance detectors, yet the physical origin of this correlation remains unclear. Here, complementary photoluminescence techniques with a full‐spectrum fit are combined to reveal the importance of vertical diffusion in governing photoluminescence response, ultimately shaping detector performance. High‐quality crystals exhibit larger vertical diffusion coefficients (up to 0.65 cm^2^ s^−1^) and lower recombination rates (down to 1.1 × 10^6^ s^−1^), leading to diffusion lengths up to 5 times greater than those in low‐quality crystals. Using one‐ and two‐photon photoluminescence microscopy, microscale defects are further visualized, with suppressed redshift and distributions throughout the bulk, in low‐quality crystals. Two‐photon diffusion mapping directly reveals how these defects hinder carrier transport. These findings establish a direct link between photoluminescence and carrier diffusion, providing a quantitative framework that connects crystal quality to charge transport and device performance in perovskite radiation detectors.

## Graphical abstract

By tracking bulk carrier diffusion both vertically and laterally, this study links photoluminescence behavior to crystal quality in CsPbBr_3_ γ‐ray detectors. Vertical diffusion governs spectral redshift, with high‐quality crystals exhibiting superior transport, while two‐photon microscopy reveals microscale defects that hinder diffusion in low‐quality crystals. These insights provide a quantitative framework for screening and optimizing crystals for radiation detection.

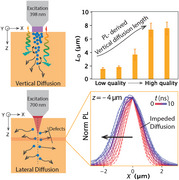

## 1 Introduction

Radiation detection of X‐rays and γ‐rays at room temperature is required in various non‐destructive technological applications, such as industrial inspection,^[^ ^1^, ^2^ ^]^ homeland security^[^ ^3^, ^4^ ^]^ and medical imaging.^[^ ^5^, ^6^ ^]^ Currently, these applications rely on semiconductors such as amorphous Se and Cd_1_₋_x_Zn_x_Te (CZT, x ≈ 0.1) single crystals. However, these materials face limitations, amorphous Se is restricted to low‐energy detection (up to 30 keV),^[^ ^7^ ^]^ while CZT suffers from high production costs.^[^ ^8^ ^]^ Since the first demonstration of a CsPbBr_3_ single‐crystal radiation detector in 2013,^[^ ^9^ ^]^ metal halide perovskites have gained attention as promising candidates for next‐generation detectors. These materials strike a balance between low‐temperature, low‐cost processing and high sensitivity across photon energies ranging from low‐energy X‐rays (≈20 keV), to medical CT energies (≈100 keV), and up to high‐energy gamma rays (>500 keV).^[^ ^10^, ^11^, ^12^ ^]^

Despite significant progress in optimizing perovskite radiation detectors, through approaches such as bias cycling^[^ ^13^ ^]^ and surface passivation,^[^ ^14^, ^15^ ^]^ high bulk crystal quality remains the foundation for achieving good device performance.^[^ ^16^ ^]^ Photoluminescence (PL) spectroscopy has been widely used as a fast, non‐invasive method for assessing crystal quality. In particular, PL lifetimes are often used as a proxy for trap‐assisted nonradiative recombination, with longer lifetimes generally interpreted as an indication of higher crystal quality.^[^ ^17^, ^18^ ^]^ Although the term “lifetime” can be physically ambiguous for intrinsic perovskites and is notoriously difficult to interpret,^[^ ^19^ ^]^ slow PL decay is commonly associated with crystals that exhibit good device performance.^[^ ^15^, ^16^, ^20^, ^21^, ^22^ ^]^ The PL peak wavelength is another property empirically reported to correlate with PL decay and crystal quality. For example, in freshly cleaved CsPbBr_3_ single crystals, a red‐shifted PL peak is statistically associated with slower PL decay and better device performance.^[^ ^23^, ^24^ ^]^ However, the underlying mechanism behind this correlation remains unclear.

Numerous spectroscopic studies have underscored the role of vertical diffusion in accelerating early PL decay and red‐shifting PL spectra in perovskite single crystals. Yang et al. applied a 1D diffusion model to extract surface recombination velocities in MAPbBr_3_ crystals from transient reflectance data.^[^ ^25^ ^]^ Wenger et al. used a similar approach to estimate trap densities and highlighted the contribution of vertical diffusion to early PL dynamics.^[^ ^26^ ^]^ As carriers diffuse away from the surface, PL originating from the bulk can undergo reabsorption before reaching the detector, resulting in a red shift in measured PL spectra. This effect has been proved using two‐photon excitation by probing depth‐selective steady‐state PL spectra, and one‐photon excitation by tracking time‐resolved PL spectral shifts.^[^ ^26^, ^27^, ^28^ ^]^ Despite strong evidence of reabsorption in perovskite single crystals, early time‐resolved photoluminescence (TRPL) simulations rarely included this spectral component. Staub et al. provided a more comprehensive model that accounts for the reabsorption by approximating carrier diffusion as a thin emissive plane moving deeper into the bulk over time.^[^ ^29^ ^]^ More recently, similar approaches have been adopted to extract vertical diffusion coefficients in both 3D and 2D perovskite thin films.^[^ ^30^, ^31^, ^32^ ^]^

Here, we present a photophysical model that explains the mechanism behind PL‐based screening of perovskite single crystals for fabricating radiation detectors. By incorporating photon reabsorption into a 1D, time‐dependent diffusion framework, we directly link PL characteristics to crystal quality in detector‐grade CsPbBr_3_. Comparisons across Bridgman‐grown crystals of varying quality reveal a clear correlation between photophysical behavior and radiation detection performance. High‐quality crystals exhibit larger vertical diffusion coefficients and longer carrier lifetimes, resulting in longer diffusion lengths that are critical for efficient charge collection. Building on this insight, we employ one‐ and two‐photon PL microscopy to map local PL variations and bulk heterogeneity at the microscale. Notably, depth‐resolved two‐photon PL maps reveal greater densities of bulk defects in low‐quality crystals that limit local carrier diffusion. These findings establish a direct connection between macroscopic PL features and detector performance and highlight the value of combining complementary PL techniques to probe charge transport and heterogeneity in three dimensions.

## 2 Results and Discussion

### 2.1 Understanding Crystal Quality from PL Spectroscopy

**Figure 1:**
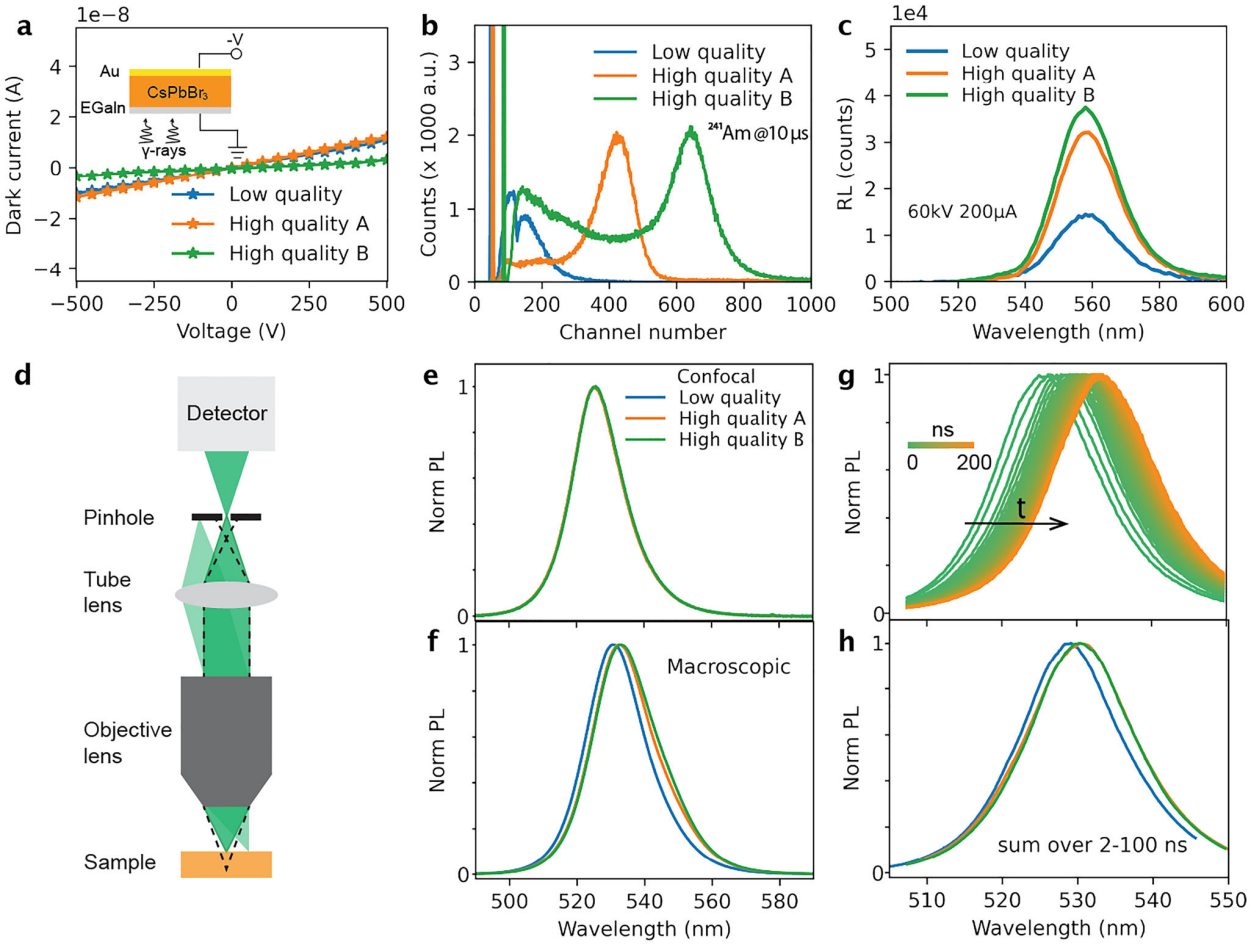
Detector and PL characteristics of CsPbBr3 crystals with varying qualities. a) *I–V* characteristic curves performed from +500  to −500 V. Inset: Schematic of device structure for radiation detection. b) ^241^Am gamma response of detectors at an applied voltage of −100 V. c) Radioluminescence spectra of CsPbBr_3_ single crystals measured in transmission configuration at 60 kV 200 µA. d) Schematic of PL collection from the sample surface using a confocal microscope. e) Confocal PL spectra of CsPbBr_3_ crystals with varying qualities collected through a 50 µm pinhole with excitation at 405 nm. f) Macroscopic PL spectra of the same quality CsPbBr_3_ crystals as in (e), excited at 405 nm with a continuous wave laser. g) Time‐resolved PL spectra of a CsPbBr_3_ crystal collected in widefield mode, excited with a pulsed laser (1.43 µJ/cm^2^/pulse and 20 kHz) at 398 nm. h) Time‐resolved PL spectra summed over 2–100 ns for CsPbBr_3_ crystals with varying qualities. All PL spectra are collected from freshly cleaved surfaces to avoid surface contamination and normalized to the maximum PL.

In this work, we compare a series of CsPbBr_3_ single crystals of varying quality, synthesized via the Bridgman method at different growth rates (Figure S1, Supporting Information). Crystals of each quality were fabricated into γ‐ray detectors to study the dark current and γ‐ray response as proxies for crystal quality. The device structure is schematically shown in **Figure** **1**a (inset), where gold paste and eutectic gallium–indium alloys (EGaIn) are used as the cathode and anode, respectively. By sweeping the voltage from +500 to −500 V, the dark current of all devices was measured (Figure 1a). All devices showed similarly low dark currents with 3.6, 10, and 12 nA at −500 V. Nonetheless, their γ‐ray responses differ considerably (Figure 1b): the low‐quality crystal cannot resolve the 59.5 keV ^241^Am γ‐response, which is well‐resolved in both high‐quality crystals with greater channel number and improved energy resolution—430 and 24% for high‐quality A, and 644 and 20% for high‐quality B, respectively. A consistent performance is seen across various γ‐ray energies (Figure S2, Supporting Information). Similarly, in their radioluminescence (RL) spectra, high‐quality crystals consistently show stronger RL signals than low‐quality ones across photon energies from 20  to 60 keV (Figure 1c; Figure S3, Supporting Information).

Since the quality of different crystals can be confirmed by their performance under radiation, we next investigated how crystal quality relates to their PL properties. All PL spectra are collected in a reflection configuration (i.e., excitation and detection from the same side) unless specified otherwise. An intriguing observation lies in the spectral differences between steady‐state PL measured using a confocal microscope and that obtained with a macroscopic setup. In the macroscopic configuration, both excitation and emission are performed over large areas, on the order of hundreds of micrometres. In the confocal setup, excitation is achieved with a diffraction‐limited laser spot (FWHM ≈270 nm; Figure S4, Supporting Information), and the emitted light is collected through a small pinhole that blocks out‐of‐focus signals both laterally and axially, as illustrated in Figure 1d.^[^ ^33^ ^]^ With a 50 µm pinhole, the optical section thickness is limited to ≈700 nm (Note S1, Supporting Information). By blocking emission from deeper regions, the normalized confocal PL spectra show negligible differences across crystals of varying quality (Figure 1e). In contrast, the macroscopic PL spectra (Figure 1f) reveal a noticeable redshift in both high‐quality crystals, consistent with previous reports. More data from different regions of interest (ROIs) can be found in Figure S5 (Supporting Information). These results suggest that the peak position of PL collected near the surface is largely insensitive to crystal quality, whereas the redshift observed in macroscopic measurements likely originates from bulk properties.

To further understand the origin of the redshift in the macroscopic PL spectra, we compared time‐resolved PL spectra of different crystals using a widefield microscope. In this setup, the sample was illuminated with a large top‐hat beam (diameter ≈150 µm), and emission was collected from a smaller region within the excitation area (Figure S6, Supporting Information). This configuration minimizes the influence of lateral carrier diffusion while still allowing vertical diffusion. As exemplified in Figure 1g, the normalized time‐resolved PL spectra of the high‐quality crystal B exhibit a gradual redshift over time. Notably, by integrating the first 100 ns of the time‐resolved data (Figure 1h; Figure S7, Supporting Information), the redshift observed in macroscopic PL spectra of high‐quality crystals is well reproduced.

**Figure 2:**
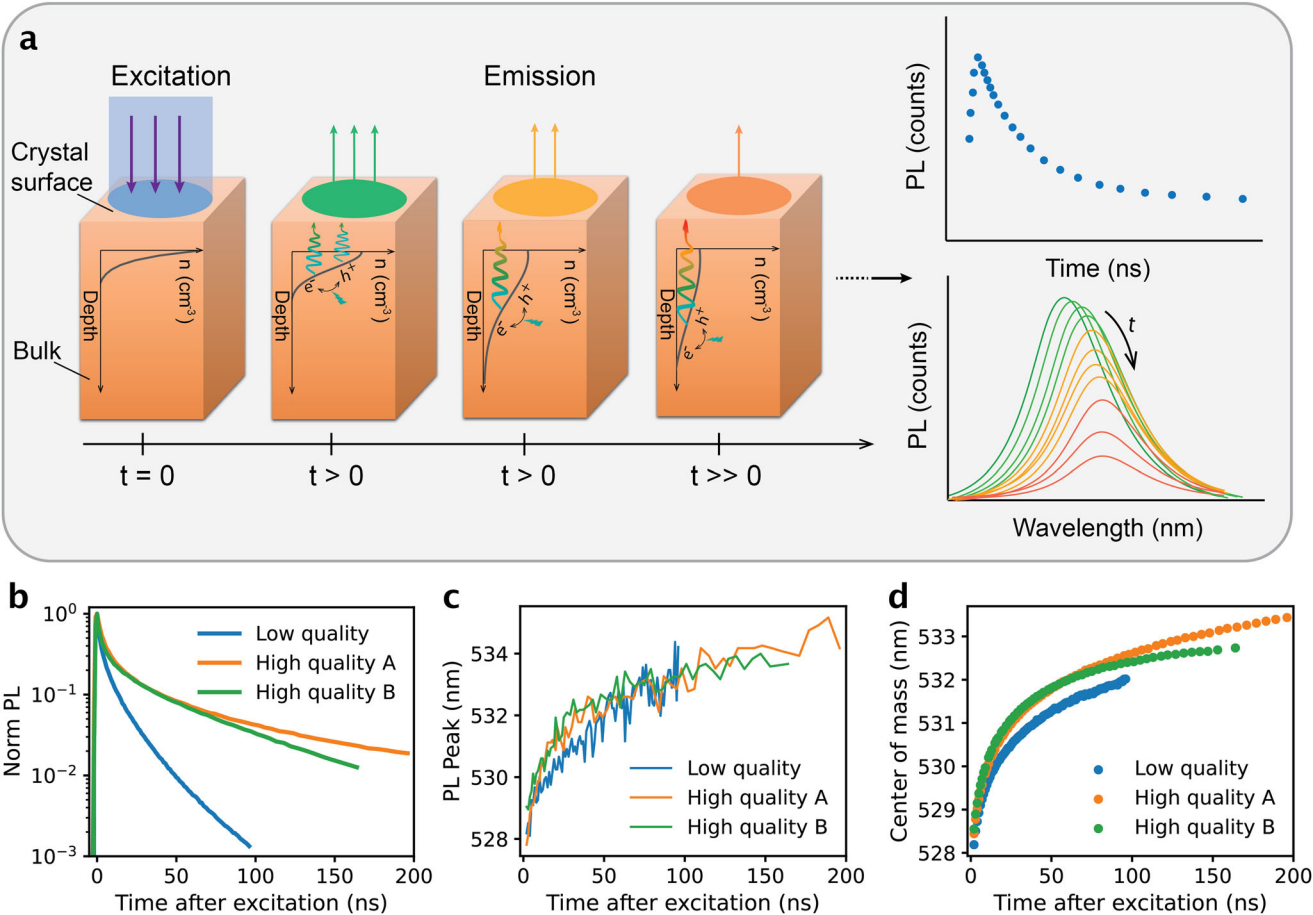
Schematic illustration and experimental results of time‐resolved PL in thick CsPbBr_3_ crystal samples. a) Schematic illustration of charge carrier distribution and PL spectra as a function of time. Upon photoexcitation (t = 0) with a widefield illumination at 398 nm, charge carriers are predominantly generated at the surface. After time zero, photogenerated charge carriers diffuse away from the surface while recombining and emitting, resulting in a red shift in the PL spectra while decaying over time. b) Time‐resolved PL decays of CsPbBr_3_ crystals with varying qualities. c) Experimental results of PL peak wavelength as a function of time. d) Experimental center of mass as a function of time, calculated between 509 and 545 nm.

To consolidate the PL results obtained from different measurements, we propose a mechanism based on photon reabsorption driven by vertical diffusion of charge carriers (**Figure** **2**a). Upon photoexcitation at a short wavelength (e.g., 398 nm), carriers are predominantly generated near the surface due to the large absorption coefficient at this wavelength. According to the Beer–Lambert law, the initial carrier distribution at t = 0 decays exponentially with depth into the crystal. Over time, this sharp profile broadens as carriers diffuse away from the surface, while simultaneously recombining and generating PL in the bulk. However, photons emitted at shorter wavelengths are likely to be reabsorbed instead of reaching the surface and being detected. This selective reabsorption leads to a time‐dependent redshift of the PL spectrum. Concurrently, the overall PL intensity decays as the carrier population diminishes. By integrating over the spectral dimension, the TRPL decay profile can be reconstructed. Therefore, selective detection of surface PL using confocal microscopy is insensitive to photon reabsorption deeper in the crystal. In contrast, macroscopic measurements and widefield microscopy can resolve the redshift in bulk PL arising from a deeper average emission plane, which directly reflects the extent of vertical carrier diffusion. In line with this mechanism, steady‐state PL spectra collected in the transmission configuration (i.e., excitation and detection from opposite sides) exhibit identical spectral shapes and peak positions (≈562 nm) across all crystal qualities and ROIs (Figure S5, Supporting Information, right panel). This behavior arises from complete self‐absorption in samples of this thickness (1 ± 0.2 mm), which masks the diffusion‐induced reabsorption, consistent with the transmitted RL spectra (Figure S3, Supporting Information).

Based on the proposed model, we compare the experimental results for different crystals, as shown in Figure 2b–d. The TRPL decays in Figure 2b show that both high‐quality crystals exhibit markedly slower PL decay (i.e., longer carrier lifetimes, τ) than the low‐quality analogue, consistent with trends reported in the literature. To track the spectral shift, a straightforward approach is to plot the PL peak wavelength versus time (Figure 2c). Although this method is often limited by noise, it still reveals a faster redshift in high‐quality crystals compared with the low‐quality crystal. Alternative metrics include the spectral center of mass (COM, calculated as intensity‐averaged wavelength) and the red‐to‐blue intensity ratio (long‐ versus short‐wavelength sides of the spectrum). The latter is compatible with PL collection based on shortpass and longpass filters, whereas the former offers a better signal‐to‐noise ratio by exploiting a wide spectral range. In all cases, the relative comparison between crystals remains consistent (Figure 2d; Figure S8, Supporting Information). As shown in Figure 2d, within the same time window, the high‐quality crystals undergo a larger redshift than the low‐quality one, indicating a faster diffusion process (i.e., larger diffusion coefficient, *D*). Given that the diffusion length scales with both the diffusion coefficient and carrier lifetime ($L_{D}\propto \sqrt{D\tau }$), the combination of fast diffusion and long carrier lifetime leads to a substantially larger diffusion length in high‐quality crystals. As a result, the emission plane resides deeper within the bulk, consistent with stronger reabsorption and further enhanced redshift in their PL spectra.

### 2.2 Quantifying Vertical Diffusion from Simulation and Fitting

Building upon our qualitative understanding of crystal quality and associated PL characteristics, we now quantify the photophysical properties of these crystals, including recombination constants, surface recombination velocities, and vertical diffusion coefficients. To simultaneously describe the PL decay dynamics and spectral shifts, we implement a numerical model accounting for first‐order trap‐assisted recombination, second‐order radiative recombination, surface recombination, vertical diffusion, and photon reabsorption effects. Within the measured time window (<200 ns), trapping and detrapping from shallow traps, which typically manifest on microsecond timescales,^[^ ^34^ ^]^ are expected to have a negligible impact on carrier dynamics and are therefore not considered. Additionally, since photon recycling has a minimal effect on PL collected in reflection mode for single crystals (Note S2, Supporting Information), it is not included in the quantitative analysis.

We start by simulating the spatiotemporal evolution of photogenerated charge carriers, which can be described by a 1D partial differential equation (PDE):

(1) $$\frac{\partial n\left(x,t\right)}{\partial t}=-k_{1}n\left(x,t\right)-k_{2}n^{2}\left(x,t\right)-k_{3}n^{3}\left(x,t\right)+D\frac{\partial^{2}n\left(x,t\right)}{\partial x^{2}}$$

where *n*(*x*, *t*) is the spatial distribution of carrier density at time *t*; *k*_1_, *k*_2_, and *k*_3_ are the trap‐assisted, radiative, and Auger recombination rates, respectively, and *D* is the diffusion coefficient. Since lead‐based halide perovskites are in general intrinsic semiconductors, electron and hole densities are treated equally (*n_e_* = *n_h_* ). Boundary conditions at the front (*x*_0_) and back (*x*_end_) surfaces are described by surface recombination velocities *S*_1_ and *S*_2_, respectively:

(2) $${\left.\frac{\partial n}{\partial t}\right|}_{x=x_{0}}=-\frac{S_{1}}{D}n\left(x_{0},t\right)$$

(3) $${\left.\frac{\partial n}{\partial t}\right|}_{x=x_{\mathrm{end}}}=-\frac{S_{2}}{D}n\left(x_{\mathrm{end}},t\right)$$

Here, we assume identical surface recombination velocities for electrons and holes but allow them to differ between the front and back surfaces. Similarly, electrons and holes are assumed to have the same diffusion coefficient for simplicity, given that their mobilities have been reported to differ by no more than a factor of two.^[^ ^35^ ^]^

Next, we simulate the PL spectra at each time point by incorporating photon reabsorption effects. Each PL spectrum is divided into small spectral segments, and for each segment, the intensity reaching the surface *x*_0_ from position *x* is calculated using the Beer‐Lambert law:

(4) $$I_{\mathrm{PL}}\left(E_{\lambda },t\right)\propto \int \mathrm{PL}\left(E_{\lambda }\right)e^{-\alpha \left(E_{\lambda }\right)x}n^{2}\left(x,t\right)\mathrm{dx},$$

where *PL*(*E*_λ_) is the intrinsic PL spectrum, approximated here using the initial PL spectrum at *t*  =  0 ns when the redshift is expected to be minimal. The absorption coefficients, *α*(*E* *_λ_*), are taken from literature values obtained by spectroscopic ellipsometry on melt‐grown CsPbBr_3_ single crystals.^[^ ^36^ ^]^ The final PL spectrum at each time point is obtained by integrating this emission over the spatial distribution of charge carriers. From these time‐dependent spectra, the PL COM can be calculated as a function of time. Calculating the overall intensity of each spectrum across all the wavelengths produces a simulated TRPL decay curve. In practice, the experimental TRPL decay at early times is broadened by convolution with the instrument response function (IRF) of the setup, leading to an apparently slower PL decay with normalization. This broadening complicates the direct comparison between simulated and experimental curves, as illustrated in Figure S9 (Supporting Information). To address this, we transform the TRPL curve, *I*(*t*), into differential decay time, defined as −2 d*t*/dln*I*(*t*).^[^ ^37^ ^]^ This quantity can then be directly compared with experimental results for extracting useful rate constants, such as the trap‐assisted recombination rate. The flowchart of the simulation procedure is summarized in Figure S10 (Supporting Information).

To examine the influence of different simulation parameters on PL decays and spectral shift, we varied all six parameters (*k*_1_, *k*_2_, *k*_3_, *D*, *S*_1_, *S*_2_) over ranges typically reported for halide perovskites.^[^ ^29^, ^38^ ^]^ As evident in Figure S11 (Supporting Information), none of the recombination rates contributes to the spectral shift, though their effects on PL decay differ: *k*_1_ governs late‐time decay (>100 ns), *k*_2_ matters only at large values (>5 × 10^−10^cm^3^ s^−1^), and *k*_3_ is negligible at the measured carrier density (1.3–1.6 × 10^17^ cm^−3^). As compared with the front‐surface recombination (*S*_1_), vertical diffusion (*D*) has a stronger effect on the spectral shift while leaving differential decay times unchanged. Finally, the back‐surface recombination velocity (*S*_2_) is negligible for large crystal thicknesses (>500 µm). We therefore set *k*_3_ and *S*_2_ to zero in subsequent simulations.

To obtain optimized simulation parameters that reliably describe the experimental results, we performed a full‐spectrum fit by wrapping the PL spectra at each time step into the fitting model. As compared with manual optimization, this approach provides more rigorous error analysis and allows direct assessment of fit quality by comparing fitted and experimental spectra. Further validation is provided in Note S3 and Figure S12 (Supporting Information).

Following the full‐spectrum fitting procedure, we extracted the parameters *k*_1_, *k*_2_, *D*, and *S*_1_ from experimental data on five crystals (low quality × 2 and high quality × 3). The corresponding fitted and experimental results are plotted in Figures S13–S17 (Supporting Information), and the complete set of fitted parameters is listed in Table S1 (Supporting Information). Parameter uncertainties are reported as one standard error (±1σ), calculated from the square roots of the diagonal elements of the covariance matrix returned by the fitting routine. The large uncertainties in *k*_2_ and *S*_1_ suggest that additional constraints, such as fluence‐dependent measurements and different surface treatments, will be required to extract these parameters independently. Nonetheless, the relatively small uncertainties in *k*_1_ and *D* indicate that these parameters are well constrained by the present approach, supporting the reliability of the fits.

**Figure 3:**
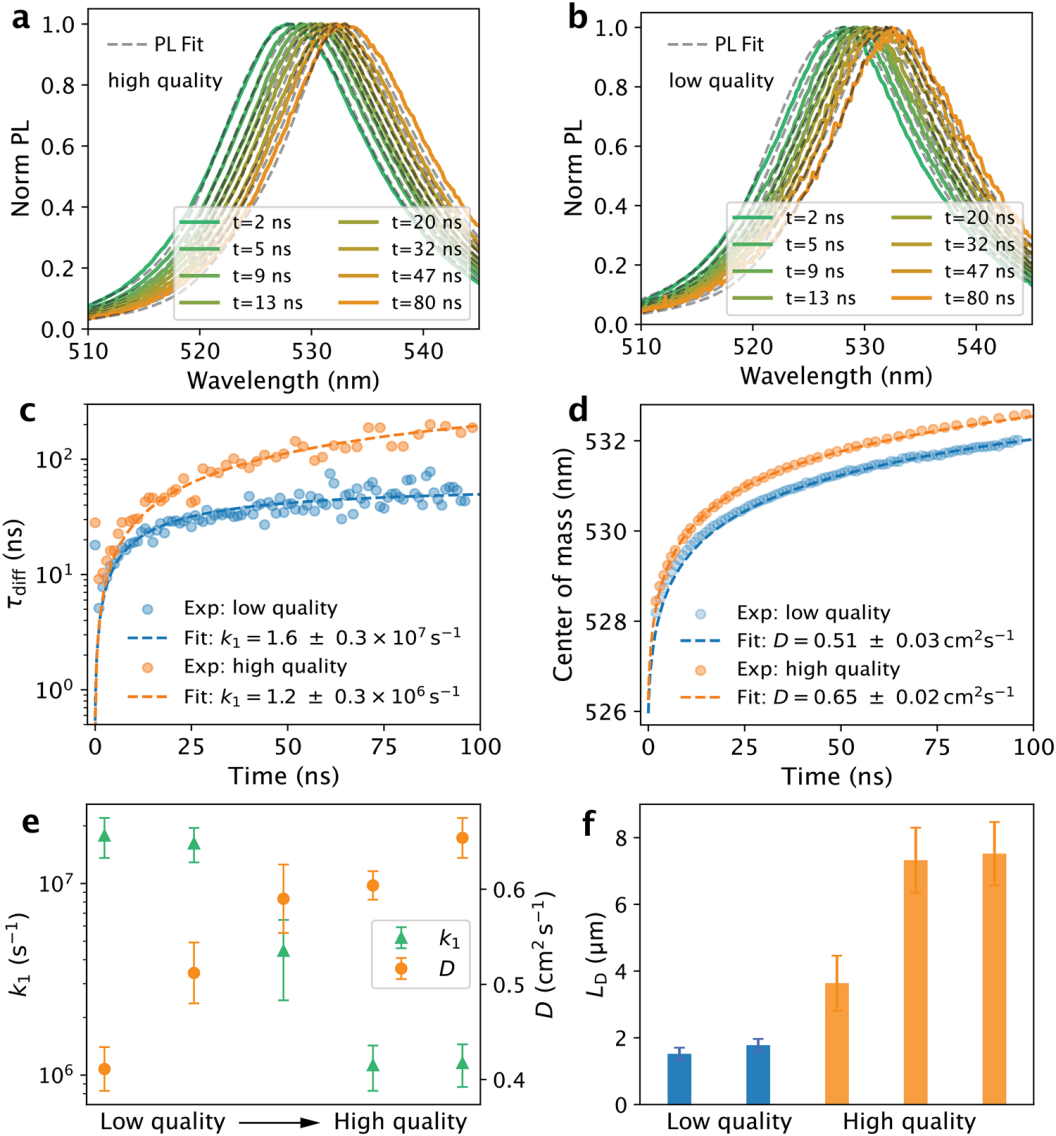
Full‐spectrum fit of spectral evolution and extracted PL properties in CsPbBr_3_ single crystals, highlighting the difference between low‐ and high‐quality crystals. a) Time‐resolved PL spectra and their fits for the high‐quality crystal. b) Time‐resolved PL spectra and their fits for the low‐quality crystal. c) Differential decay times and their fits. d) Center of mass as a function of time and simulated fits. e) Trap‐assisted recombination rates (*k*_1_, green triangles) and vertical diffusion coefficients (*D*, orange dots) fitted for all crystals. f) Vertical diffusion lengths (*L*_D_) calculated from $\sqrt{D/k_{1}}$ for all crystals. The error bar represents the one standard error (±1σ) from the fits.

Next, we compare the simulated TRPL results with the experimental data of both high‐quality and low‐quality crystals. **Figure** **3**a,b shows normalized PL spectra and their corresponding fits at different times, where the spectral shifts in experiments are well reproduced using the fitted parameters, confirming the quality of the fitting. In their differential decay times (Figure 3c), the main difference between the two crystals arises from their trap‐assisted recombination rates (*k*_1_). While radiative and Auger recombination are more intrinsic material properties, trap‐assisted recombination varies strongly with defect concentration.^[^ ^39^ ^]^ Accordingly, the one‐order‐of‐magnitude smaller *k*_1_, in the high‐quality crystal (1.2 ± 0.3 × 10^6^ s^−1^) compared with the low‐quality one (1.6 ± 0.3 × 10^7^ s^−1^) indicates a much lower density of deep traps. The vertical diffusion coefficients further reflect these differences (Figure 3d): 0.65 ± 0.02 cm^2^ s^−1^ for the high‐quality crystal and 0.51 ± 0.03 cm^2^ s^−1^ for the low‐quality one. From these values, we estimate diffusion lengths ($L_{D}\propto \sqrt{D\tau }$) of 7.4 µm in the high‐quality crystals, ≈4 times longer than that in the low‐quality crystal (1.8 µm). This trend is consistent across all 5 crystals, as shown in Figure 3e,f, quantitatively confirming the superior vertical charge transport properties in the high‐quality crystals.

### 2.3 Visualizing Heterogeneity from PL Microscopy

**Figure 4:**
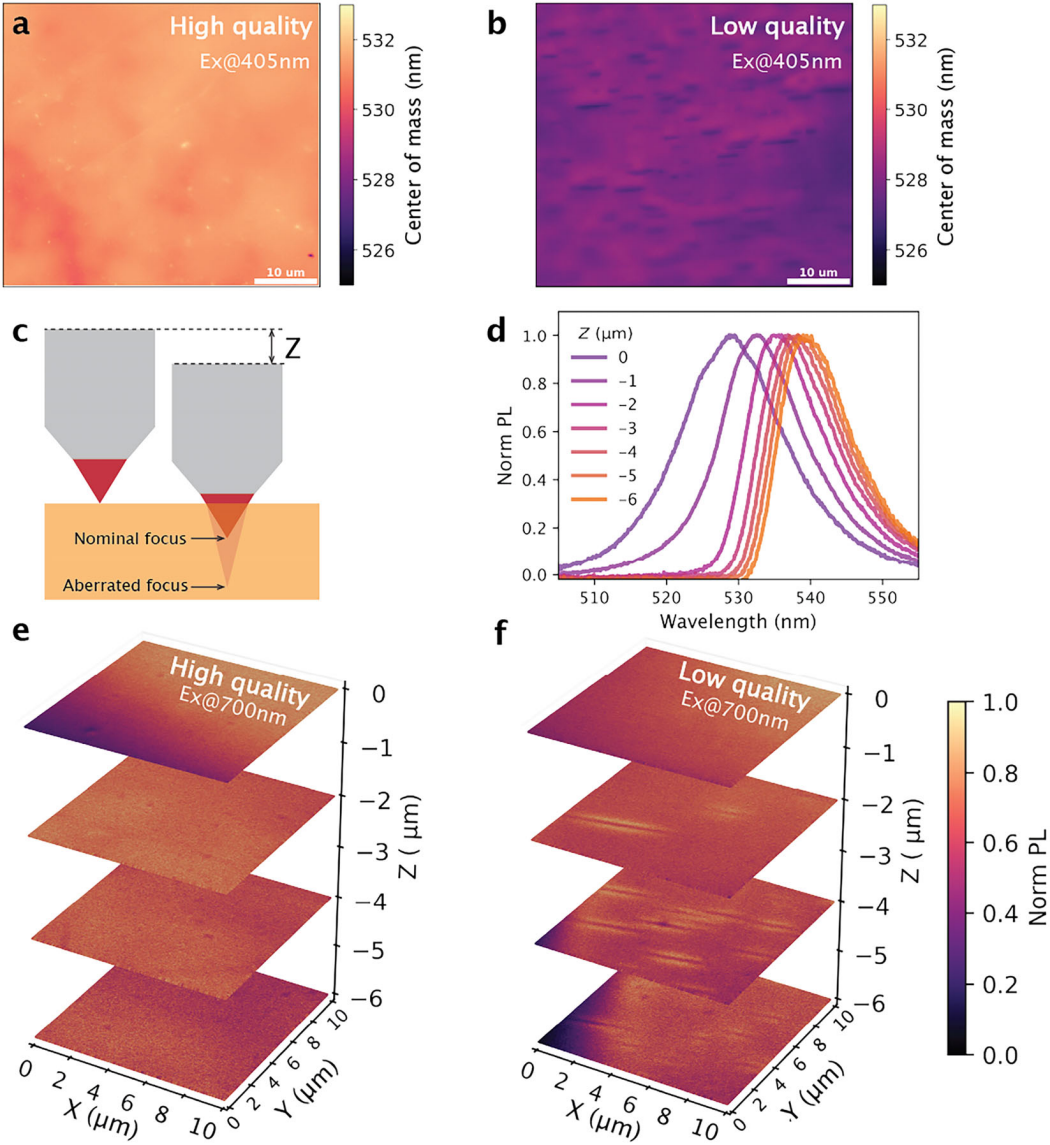
Microscopic PL results of CsPbBr_3_ single crystals reveal buried defective sites in the low‐quality crystal. a) PL center of mass map of a high‐quality CsPbBr_3_ crystal and b) a low‐quality CsPbBr_3_ crystal, measured with a widefield microscope (λ_ex_ = 405 nm). c) Schematic of depth‐dependent two‐photon PL microscopy, with depth Z defined as the displacement of the objective from its position focusing on the crystal surface. Aberration effects are discussed in Note S4 and Figure S19 (Supporting Information). d) PL spectra collected with two‐photon excitation at different depths, showing a redshift at greater depths due to self‐reabsorption. Normalized PL intensity map of a high‐quality CsPbBr_3_ crystal in e) and a low‐quality CsPbBr_3_ crystal in f) at different depths, using two‐photon excitation. All two‐photon measurements were excited at 700 nm (1000 µJ cm^−2^ pulse) and collected through a 75 µm pinhole. [Correction added on October 20, 2025, after first online publication: Caption of Figure 4 has been updated.]

To further assess the crystal quality at the microscale, we used widefield hyperspectral microscopy to capture spectral information from the crystal surface. **Figure** **4**a,b shows the PL COM maps for the high‐quality and low‐quality crystals, respectively. Notably, the low‐quality crystal exhibits an average 4‐nm blueshift compared to the high‐quality analogue, consistent with the trend observed in the macroscopic PL spectra. In addition to the overall smaller COM, numerous stripe‐like features are visible in the low‐quality crystal (Figure 4b), with a COM emission wavelength as low as 521 nm. Since these stripes are absent in the white‐light reflection images of the same ROIs as shown in Figure S18 (Supporting Information), we can rule out the origin of the optical effect due to surface morphology. We hypothesize that these stripes represent defects that impede charge carriers, hindering their diffusion and suppressing the reabsorption effect.

To further examine these defects in the bulk, we used PL microscopy with two‐photon (2P) excitation. By taking advantage of the small 2P absorption coefficients, this technique offers deep optical penetration that allows us to study the spatial and temporal evolution of PL signals tens of micrometres beneath the crystal surface (Figure 4c). A femtosecond laser with a sub‐bandgap wavelength of 700 nm was used to excite the crystal from the surface to depths of tens of micrometres, by focusing the objective into the crystal over a nominal distance (*Z*). Note that the actual focal position is deeper than the nominal one due to aberration effects caused by the refractive index mismatch between air (*n*  =  1) and the perovskite crystals (*n* ≈ 2.2).^[^ ^40^ ^]^ These aberrations cause the depth of focus to scale linearly with *Z*, leading to an ill‐defined focal position as the light intensity distribution spreads out with increasing depth.^[^ ^41^, ^42^ ^]^ For clarity, the nominal focal position, *Z*, is used to label the depth of each measurement in the main text, with a more detailed discussion of the actual focal position provided in Note S4 and Figure S19 (Supporting Information). Figure 4d shows the 2P PL spectra collected through a 75 µm pinhole at different depths. The clear redshift in the spectra at larger depths signals the reabsorption effect, confirming that the collected PL originates from the bulk.

By raster scanning the laser excitation and collecting emission over the ROI, we obtained PL intensity maps of different crystals that visualize bulk morphology at different depths. As shown in Figure 4e,f, both crystals show good homogeneity at the surface. Beyond the surface, the stripe‐like features seen in Figure 4b are again revealed in the bulk of the low‐quality crystal (Figure 4f) with varying distribution at different depths, while remaining absent in the high‐quality crystal (Figure 4e). These features appear as dark stripes sandwiched between two bright ones, indicating enhanced radiative recombination due to accumulated carriers in the vicinity of defective sites as a result of impeded diffusion. We confirm the consistency of these differences in the bulk by mapping multiple ROIs on both low‐ and high‐quality crystals (Figures S20–S25, Supporting Information). When such defective sites are presented on the surface, they can be further confirmed by scanning electron microscopy. The secondary electron image of a cleaved low‐quality crystal reveals microscale, elongated stripe‐like features, in agreement with the PL maps (Figure S26, Supporting Information). While secondary electron images alone cannot confirm the nature of these features, our optical studies reveal their presence within the bulk and motivate future investigations with complementary methods to verify their origin.

### 2.4 Tracking Lateral Diffusion in the Bulk

**Figure 5:**
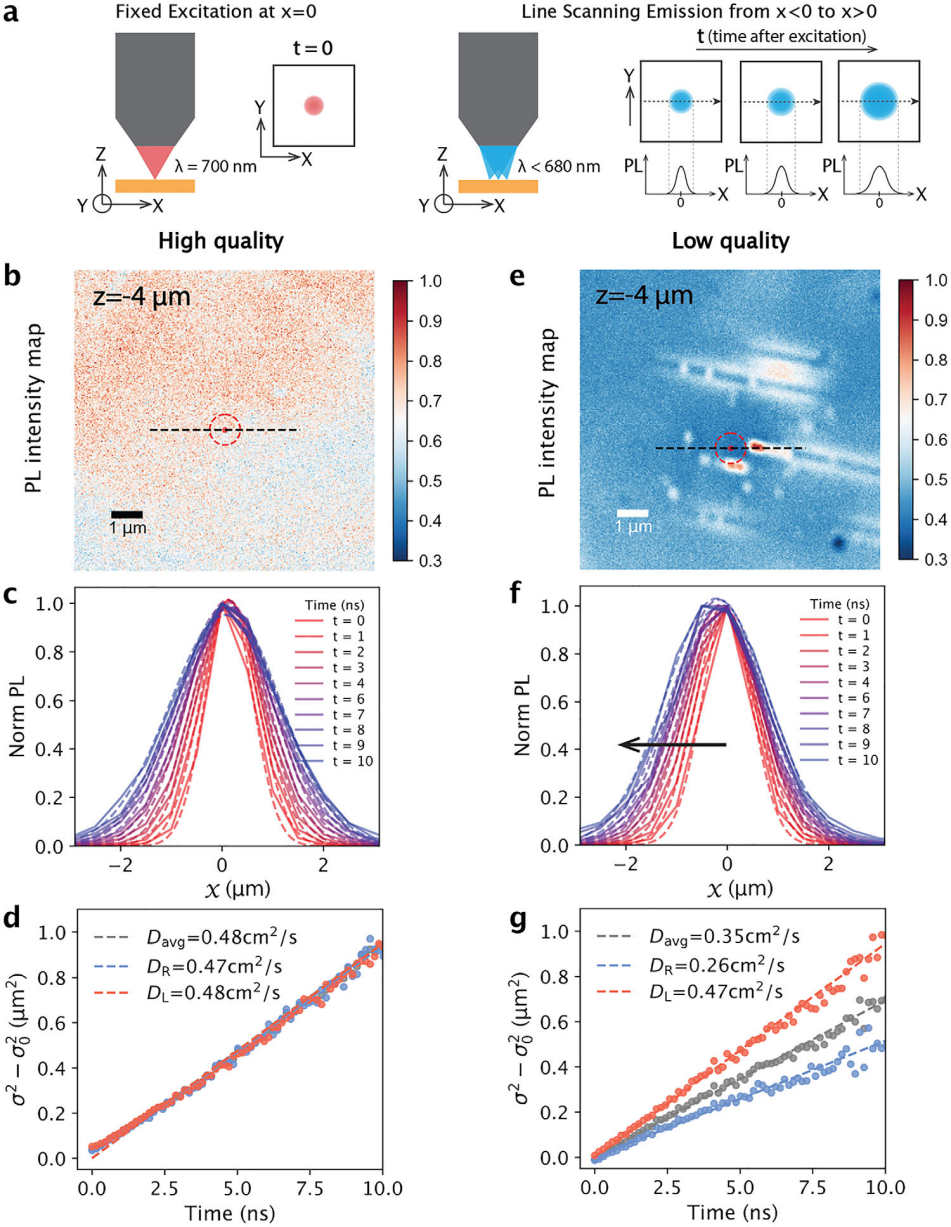
Effect of bulk defective sites on local charge transport in CsPbBr_3_ crystals. a) Schematic of the line scan approach to track the lateral diffusion properties with a fixed 2P excitation. Normalized PL intensity map highlighting the region of interest in b) a high‐quality CsPbBr_3_ crystal and e) a low‐quality CsPbBr_3_ crystal. c,f) Spatial PL profiles from diffusion measurements (solid lines) and fits (dotted lines) at different times, showing asymmetric carrier distribution broadening across defects in f) and symmetric broadening in the absence of defects in c). d,g) Evolution of squared broadening (σ^2^) as a function of time, extracted from Gaussian TRPL profiles for diffusion to the left (red) and right (blue) in c,f).

To directly visualize how these defects affect the charge carrier diffusion in the bulk, we performed confocal PL diffusion measurement with 2P excitation. In the PL diffusion measurement, the sample is excited by a diffraction‐limited laser beam at a fixed position (FWHM ≈515 nm, Figure S27, Supporting Information). As the charge carriers diffuse away from the excitation spot, the corresponding PL profile expands over time. The temporal evolution of this PL expansion is tracked by performing a line scan along the excitation spot with a step size of 500 nm, as illustrated in **Figure** **5**a. During the line scan, the TRPL curve at each position *x* along the line of interest is collected via a time‐correlated single photon counting measurement.^[^ ^43^, ^44^ ^]^ With the TRPL curves at different positions along this line, we reconstruct the corresponding spatial PL profile at a specific time after the excitation.

In Figure 5b–g, we compare 2P PL diffusion results between the high‐quality and low‐quality crystals with a nominal focus at *z* = − 4 µm. More diffusion results at different depths and ROIs are provided in Figures S28 and S29 (Supporting Information). In the high‐quality crystal, the broadening of the PL profile reveals a symmetrical diffusion behavior (Figure 5c), consistent with the good homogeneity of the region (Figure 5b). To quantify the diffusion coefficients, the PL profile at each time is fitted by a Gaussian function. The squared standard deviation (σ^2^) of each Gaussian fit is plotted as a function of time (*t*) in Figure 5d. The linear relationship between σ^2^ and *t* indicates a classical diffusive behavior,^[^ ^45^ ^]^ that allows the determination of diffusion coefficient (*D*) from a linear mean‐squared displacement model, where σ(*t*)^2^ = σ(0)^2^  + 2*Dt*.^[^ ^46^ ^]^ In this case, we found the diffusion coefficients to be 0.48 cm^2^ s^−1^. In the low‐quality crystal (Figure 5f), the PL profile immediately after the photoexcitation (*t*  =  0) remains symmetric, reflecting the shape of the excitation beam. However, within the next few nanoseconds, the PL profile expands asymmetrically, with noticeably slower diffusion toward the right side, where defective sites are present. This provides direct evidence that microscale defects impede charge carrier diffusion in the bulk of the crystal. By separately fitting the PL broadening on the left (*x* < 0) and right (*x* > 0) regions (Figure 5g), we obtain diffusion coefficients of 0.47 cm^2^/s and 0.26 cm^2^/s for the left and right sides, respectively. These results reveal the detrimental impact of these defects on charge transport in the bulk of the crystal, which inevitably limits the device performance of the detectors.

## 3 Conclusion

In conclusion, we addressed the unclear link between single crystal quality and macroscopic PL properties—important indicators for screening materials in radiation detection. We first confirmed CsPbBr_3_ crystal quality based on their γ‐ray detector performance, then used complementary PL techniques to probe different aspects of these crystals. Surface‐limited confocal PL spectra showed PL peak positions independent of crystal quality, while widefield TRPL revealed a gradual redshift over time. Integration over 100 ns reproduced the redshift observed in steady‐state macroscopic PL. To consolidate these findings, we proposed a vertical diffusion model that explains the PL behavior across crystals of varying quality. By combining a 1D diffusion model with photon reabsorption, we performed a full‐spectrum fit to extract vertical diffusion coefficients and estimate diffusion lengths from experimental data. Our results show that high‐quality crystals exhibit enhanced PL redshift, driven by a larger vertical diffusion coefficient (0.6–0.65 cm^2^ s^−1^), compared to 0.4–0.5 cm^2^ s^−1^ in low‐quality crystals. Together with their much longer lifetimes, high‐quality crystals therefore exhibit notably longer vertical diffusion lengths—crucial for efficient charge collection and consistent with observed device performance.

Building on this understanding, we used 1P and 2P PL microscopy to examine the structure from surface to bulk. Low‐quality crystals exhibited additional microscale sites with suppressed redshift, distributed across various depths within the bulk. Finally, 2P diffusion measurements enabled us to directly visualize how these local defective sites limit charge transport in the bulk of the crystal. By linking PL characteristics to spatially resolved diffusion dynamics, this work enables the identification of performance‐limiting regions, such as microscale defective sites, that can now be targeted for elimination through improved synthesis or defect passivation. Overall, these insights establish a clear link between single crystal quality and PL via carrier diffusion, providing a fundamental basis for the rational design of high‐performance radiation detectors by guiding material selection and engineering toward structures with enhanced carrier transport.

## 4 Experimental Section

### Synthesis and Crystal Growth

Chemically in‐house synthesized and purified CsBr and PbBr_2_ precursors were used to prepare CsPbBr_3_ bulk.^[^ ^24^ ^]^ The initial precursor materials were mixed in a 1:1 amount, sealed in vacuum quartz tubes, heated to 650 °C for 8 h, kept at that temperature for another 24 h, and then allowed to cool down to room temperature. After trimming the resultant bulk ingots to eliminate the tip and tail parts, consecutive vertical Bridgman growth was subjected to them for further purification. Crystals of varying grades were produced using Bridgman growth at translation rates of 3 , 2 , and 1 mm h^−1^. The crystal tip and heal were excised after each growth phase to minimize the incorporation of contaminants. Three samples designated as low quality (3 mm h^−1^), high quality A (2 mm h^−1^), and high quality B (1 mm h^−1^) grown at different rates were used for characterization in this study.

### Device Fabrication and Measurement

CsPbBr_3_ single crystals were fabricated into detectors of different thicknesses (≈1.5 to 2 mm). Detectors were fabricated manually by using gold paste as a cathode and EGaIn as an anode material connected with copper wire to study the dark current and gamma ray response. The dark current of all three devices was acquired as the voltage swept from +500 to −500 V. γ‐ray spectra were acquired using an eV‐550 preamplifier, an AMETEK 572A amplifier, a 556 AMETEK high‐voltage power supply, and an ASPEC‐927 multichannel analyzer. The ^241^Am gamma response of the detectors was collected at a voltage of −100 V, shaping time of 10 µs, and a gain of 80.

### Radioluminescence Measurement

Radioluminescence spectra were measured in the transmission configuration using the Edinburgh Instruments XS1 system coupled to the Edinburgh Instruments FLS1000 fluorescence spectrometer. The X‐ray source was a 12‐W tungsten MagPro X‐ray imaging source from MOXTEK. X‐ray tube voltage and current were set as 60 kV 200 µA, 40 kV 300 µA, and 20 kV 600 µA.

### Macroscopic Photoluminescence

Macroscopic PL spectra of freshly cleaved crystals were acquired in reflection or transmission mode using a Teledyne Princeton Instruments HRS‐500‐SS spectrograph equipped with a Pixis 1024BRX detector. Excitation was provided by a continuous‐wave 405 nm laser (CNI MGL‐III‐405, 500 mW), focused onto the sample surface with a 1/e beam diameter of ≈250 µm. To suppress residual laser light and isolate the PL signal, a 420 nm longpass dichroic mirror and a 425 nm longpass filter were employed.

### One‐Photon Confocal Photoluminescence

Steady‐state confocal PL spectra of freshly cleaved crystals were measured using a confocal microscope setup (PicoQuant, MicroTime 200) and an EMCCD spectrograph (Kymera 193i, Oxford Instruments). A 405 nm laser was focused onto the sample through an air objective (100×, 0.9 NA). The PL signal was collected by the same objective and subsequently separated from the excitation light using a 405 nm longpass dichroic mirror (Z405RDC, Chroma) and a 450 nm longpass filter. The filtered emission was then directed through a 50 µm pinhole before being focused onto the spectrograph for spectrum collection.

### Widefield Hyperspectral Photoluminescence

Widefield hyperspectral PL measurements were performed using the Photon etc. IMA system with 100× magnification. For steady‐state hyperspectral PL mapping, a continuous‐wave 405 nm laser was used as the excitation source. Emission was directed onto a temperature‐controlled CMOS camera (Hamamatsu ORCA‐Flash 4.0 V3 sCMOS) via a volume Bragg grating. By sweeping the angle of the grating relative to the incident light, PL spectra from each pixel within the ROI were acquired with a 2 nm spectral step size. For time‐resolved PL measurements, a pulsed 398 nm laser operating at a repetition rate of 20 kHz and a fluence of 1.43 µJ cm^−2^ was used as the excitation source. The emission was collected by fixing the volume Bragg grating at a specific wavelength to record spectra over a limited range. PL spectra at various time delays after excitation were captured using an intensified electron‐multiplied CCD camera (emICCD, PI‐MAX4, Princeton Instruments) with a gate width of 1 ns. Acquired data were post‐processed using Python. All samples were measured at room temperature.

### Two‐Photon Photoluminescence Mapping and Diffusion

PL maps and diffusion measurements with two‐photon excitation were performed using a confocal microscope setup (PicoQuant, MicroTime 200). For two‐photon excitation, a 700‐nm (below the CsPbBr_3_ bandgap) pulsed laser (SpectraPhysics InSight DS+, ≈100 fs pulse width) was focused through an air objective (100x, 0.8 NA) with a repetition rate of 5 MHz and a fluence of 1000 µJ cm^−2^. A 670 nm shortpass dichroic mirror (T670spxrxt, Chroma) and a 680 nm shortpass filter were used to separate the emission signals from the excitation sources. The filtered emission was then directed through a 75 µm pinhole before being focused onto a photomultiplier detector (PicoQuant, Hybrid PMA) for time‐correlated single photon counting. To obtain the PL intensity maps, both excitation and emission were scanned pixel‐by‐pixel over the ROI using a Galvano scanner. In diffusion measurements, the emission signals were scanned pixel‐by‐pixel using a Galvano scanner, while the excitation source reached the sample through another path to remain at a fixed position. The FWHM of the excitation spot size is ≈515 nm.

## Conflict of Interest

SDS is a cofounder of Clarity Sensors Limited. MGK is a cofounder of Actinia Inc.

## Author Contributions

S.D.S. and Z.W. conceived the project. Z.W. performed the macroscopic, confocal, and two‐photon photoluminescence microscopy measurements, all photoluminescence data analysis, and the numerical simulations. K.S.B. prepared the CsPbBr_3_ single crystals and γ‐ray detectors and carried out the device measurements. C.M. conducted the hyperspectral photoluminescence microscopy experiments. M.D. and C.M. developed the setup for time‐resolved hyperspectral photoluminescence measurements and supported data interpretation. C.S.H. and Z.W. performed the radioluminescence measurements. L.P. performed SEM measurements. M.G.K. supervised K.S.B. S.D.S. supervised C.M., M.D., and Z.W. Z.W. wrote the initial draft, and all authors contributed to revising and editing the manuscript.

## Supporting information



Supporting Information

## Data Availability

The data that support the findings of this study are openly available in Apollo at https://doi.org/10.17863/CAM.121576, reference number 47.

## References

[1] X. Duan , J. Cheng , L. Zhang , Y. Xing , Z. Chen , Z. Zhao , Nucl. Instrum. Methods Phys. Res. Sect. Accel. Spectrometers Detect. Assoc. Equip. 2009, 598, 439.

[2] R. P. Haff , N. Toyofuku , Sens. Instrum. Food Qual. Saf. 2008, 2, 262.

[3] G. Harding , Radiat. Phys. Chem. 2004, 71, 869.

[4] P. M. Johns , J. C. Nino , J. Appl. Phys. 2019, 126, 040902.

[5] M. Spahn , Nucl. Instrum. Methods Phys. Res. Sect. Accel. Spectrometers Detect. Assoc. Equip. 2013, 731, 57.

[6] F. Arfelli , M. Assante , V. Bonvicini , A. Bravin , G. Cantatore , E. Castelli , L. D. Palma , M. D. Michiel , R. Longo , A. Olivo , S. Pani , D. Pontoni , P. Poropat , M. Prest , A. Rashevsky , G. Tromba , A. Vacchi , E. Vallazza , F. Zanconati , Phys. Med. Biol. 1998, 43, 2845. 9814522 10.1088/0031-9155/43/10/013

[7] A. Datta , Z. Zhong , S. Motakef , Sci. Rep. 2020, 10, 20097. 33208782 10.1038/s41598-020-76647-5 PMC7676260

[8] Y. He , I. Hadar , M. G. Kanatzidis , Nat. Photonics 2022, 16, 14.

[9] C. C. Stoumpos , C. D. Malliakas , J. A. Peters , Z. Liu , M. Sebastian , J. Im , T. C. Chasapis , A. C. Wibowo , D. Y. Chung , A. J. Freeman , B. W. Wessels , M. G. Kanatzidis , Cryst. Growth Des. 2013, 13, 2722.

[10] Y. He , L. Matei , H. J. Jung , K. M. McCall , M. Chen , C. C. Stoumpos , Z. Liu , J. A. Peters , D. Y. Chung , B. W. Wessels , M. R. Wasielewski , V. P. Dravid , A. Burger , M. G. Kanatzidis , Nat. Commun. 2018, 9, 1609. 29686385 10.1038/s41467-018-04073-3 PMC5913317

[11] J. Peng , C. Q. Xia , Y. Xu , R. Li , L. Cui , J. K. Clegg , L. M. Herz , M. B. Johnston , Q. Lin , Nat. Commun. 2021, 12, 1531. 33750768 10.1038/s41467-021-21805-0 PMC7943776

[12] Y. He , M. Petryk , Z. Liu , D. G. Chica , I. Hadar , C. Leak , W. Ke , I. Spanopoulos , W. Lin , D. Y. Chung , B. W. Wessels , Z. He , M. G. Kanatzidis , Nat. Photonics 2021, 15, 36.

[13] J. T. Tisdale , M. Yoho , H. Tsai , S. Shrestha , K. Fernando , J. K. Baldwin , S. Tretiak , D. Vo , W. Nie , Adv. Opt. Mater. 2020, 8, 2000233.

[14] B. Yang , W. Pan , H. Wu , G. Niu , J.‐H. Yuan , K.‐H. Xue , L. Yin , X. Du , X.‐S. Miao , X. Yang , Q. Xie , J. Tang , Nat. Commun. 2019, 10, 1989. 31040278 10.1038/s41467-019-09968-3 PMC6491557

[15] L. Zhao , Z. Shi , Y. Zhou , X. Wang , Y. Xian , Y. Dong , O. Reid , Z. Ni , M. C. Beard , Y. Yan , J. Huang , Nat. Photonics 2024, 18, 250.

[16] Y. Hua , G. Zhang , X. Sun , P. Zhang , Y. Hao , Y. Xu , Y. Yang , Q. Lin , X. Li , Z. Zhai , F. Cui , H. Liu , J. Liu , X. Tao , Nat. Photonics 2024, 18, 870.

[17] D. Shi , V. Adinolfi , R. Comin , M. Yuan , E. Alarousu , A. Buin , Y. Chen , S. Hoogland , A. Rothenberger , K. Katsiev , Y. Losovyj , X. Zhang , P. A. Dowben , O. F. Mohammed , E. H. Sargent , O. M. Bakr , Science 2015, 347, 519. 25635092 10.1126/science.aaa2725

[18] A. A. Zhumekenov , M. I. Saidaminov , M. A. Haque , E. Alarousu , S. P. Sarmah , B. Murali , I. Dursun , X.‐H. Miao , A. L. Abdelhady , T. Wu , O. F. Mohammed , O. M. Bakr , ACS Energy Lett. 2016, 1, 32.

[19] A. R. Bowman , S. D. Stranks , PRX Energy 2023, 2, 022001.

[20] L. Zhao , Y. Zhou , Z. Shi , Z. Ni , M. Wang , Y. Liu , J. Huang , Nat. Photonics 2023, 17, 315.

[21] Z. Ni , L. Zhao , Z. Shi , A. Singh , J. Wiktor , M. O. Liedke , A. Wagner , Y. Dong , M. C. Beard , D. J. Keeble , J. Huang , Adv. Mater. 2024, 36, 2406193. 10.1002/adma.202406193 39003617

[22] N. Shen , T. Gao , X. Ouyang , K. S. Bayikadi , Z. Duan , B. Xiao , X. He , Y. Wang , H. Qin , Q. Sun , L. Wang , Y. Lai , X. Liu , R. Ren , M. G. Kanatzidis , Y. He , ACS Photonics 2024, 11, 3662.

[23] L. Pan , I. R. Pandey , A. Miceli , V. V. Klepov , D. Y. Chung , M. G. Kanatzidis , Adv. Opt. Mater. 2023, 11, 2202946.

[24] D. Y. Chung , W. Lin , M. Unal , Q. V. Phan , I. R. Pandey , R. Vitt , Y. He , M. G. Kanatzidis , Cryst. Growth Des. 2024, 24, 9590.

[25] Y. Yang , Y. Yan , M. Yang , S. Choi , K. Zhu , J. M. Luther , M. C. Beard , Nat. Commun. 2015, 6, 7961. 26245855 10.1038/ncomms8961 PMC4918347

[26] B. Wenger , P. K. Nayak , X. Wen , S. V. Kesava , N. K. Noel , H. J. Snaith , Nat. Commun. 2017, 8, 590. 28928482 10.1038/s41467-017-00567-8 PMC5605602

[27] Y. Fang , H. Wei , Q. Dong , J. Huang , Nat. Commun. 2017, 8, 14417. 28220791 10.1038/ncomms14417 PMC5321765

[28] T. Yamada , Y. Yamada , Y. Nakaike , A. Wakamiya , Y. Kanemitsu , Phys. Rev. Appl. 2017, 7, 014001.

[29] F. Staub , I. Anusca , D. C. Lupascu , U. Rau , T. Kirchartz , J. Phys. Mater. 2020, 3, 025003.

[30] C. Cho , S. Feldmann , K. M. Yeom , Y.‐W. Jang , S. Kahmann , J.‐Y. Huang , T. C.‐J. Yang , M. N. T. Khayyat , Y.‐R. Wu , M. Choi , J. H. Noh , S. D. Stranks , N. C. Greenham , Nat. Mater. 2022, 21, 1388. 36396960 10.1038/s41563-022-01395-y

[31] J. Du , M. Righetto , M. Kober‐Czerny , S. Yan , K. A. Elmestekawy , H. J. Snaith , M. B. Johnston , L. M. Herz , Adv. Funct. Mater. 2025, 35, 2421817.

[32] Y. Yuan , G. Yan , S. Akel , U. Rau , T. Kirchartz , Sci. Adv. 2025, 11, adt1171. 10.1126/sciadv.adt1171 PMC12002118 40238866

[33] Z. Wei , M. Dubajic , C. Chosy , S. Kahmann , S. D. Stranks , Nat. Rev. Methods Primer 2025, 5, 37.

[34] Y. Yuan , G. Yan , C. Dreessen , T. Rudolph , M. Hülsbeck , B. Klingebiel , J. Ye , U. Rau , T. Kirchartz , Nat. Mater. 2024, 23, 391. 38195863 10.1038/s41563-023-01771-2 PMC10917677

[35] Y. He , Z. Liu , K. M. McCall , W. Lin , D. Y. Chung , B. W. Wessels , M. G. Kanatzidis , Nucl. Instrum. Methods Phys. Res. Sect. Accel. Spectrometers Detect. Assoc. Equip. 2019, 922, 217.

[36] M. C. Brennan , D. M. Krein , E. Rowe , C. L. McCleese , L. Sun , K. G. Berry , P. R. Stevenson , M. A. Susner , T. A. Grusenmeyer , MRS. Commun 2024, 14, 900.

[37] T. Kirchartz , J. A. Márquez , M. Stolterfoht , T. Unold , Adv. Energy Mater. 2020, 10, 1904134.

[38] J. M. Richter , M. Abdi‐Jalebi , A. Sadhanala , M. Tabachnyk , J. P. H. Rivett , L. M. Pazos‐Outón , K. C. Gödel , M. Price , F. Deschler , R. H. Friend , Nat. Commun. 2016, 7, 13941. 28008917 10.1038/ncomms13941 PMC5196482

[39] S. Nagane , S. Macpherson , M. A. Hope , D. J. Kubicki , W. Li , S. D. Verma , J. Ferrer Orri , Y.‐H. Chiang , J. L. MacManus‐Driscoll , C. P. Grey , S. D. Stranks , Adv. Mater. 2021, 33, 2102462. 34219285 10.1002/adma.202102462 PMC11468984

[40] G. Ermolaev , A. P. Pushkarev , A. Zhizhchenko , A. A. Kuchmizhak , I. Iorsh , I. Kruglov , A. Mazitov , A. Ishteev , K. Konstantinova , D. Saranin , A. Slavich , D. Stosic , E. S. Zhukova , G. Tselikov , A. Di Carlo , A. Arsenin , K. S. Novoselov , S. V. Makarov , V. S. Volkov , Nano Lett. 2023, 23, 2570. 36920328 10.1021/acs.nanolett.2c04792

[41] N. J. Everall , Appl. Spectrosc. 2000, 54, 773.

[42] D. R. Ceratti , Y. Rakita , L. Cremonesi , R. Tenne , V. Kalchenko , M. Elbaum , D. Oron , M. A. C. Potenza , G. Hodes , D. Cahen , Adv. Mater. 2018, 30, 1706273. 10.1002/adma.201706273 29328524

[43] G. M. Akselrod , F. Prins , L. V. Poulikakos , E. M. Y. Lee , M. C. Weidman , A. J. Mork , A. P. Willard , V. Bulović , W. A. Tisdale , Nano Lett. 2014, 14, 3556. 24807586 10.1021/nl501190s

[44] C. Stavrakas , G. Delport , A. A. Zhumekenov , M. Anaya , R. Chahbazian , O. M. Bakr , E. S. Barnard , S. D. Stranks , ACS Energy Lett. 2020, 5, 117. 32055687 10.1021/acsenergylett.9b02244 PMC7009023

[45] D. W. deQuilettes , R. Brenes , M. Laitz , B. T. Motes , M. M. Glazov , V. Bulović , ACS Photonics 2022, 9, 110.

[46] N. N. Wong , S. K. Ha , K. Williams , W. Shcherbakov‐Wu , J. W. Swan , W. A. Tisdale , J. Chem. Phys. 2022, 157, 104201. 36109245 10.1063/5.0100075

[47] Z. Wei , K. S. Bayikadi , C. Mamak , M. Dubajic , C. S. Huang , L. Pan , M. G. Kanatzidis , S. D. Stranks , Apollo ‐ University of Cambridge Repository 2025, 10.17863/CAM.121576

