# Supplementary material for: Carrier Diffusion Links Single Crystal Quality and Photoluminescence in Halide Perovskite Radiation Detectors
Source: Adv Mater. 2025 Oct 16;38(4):e12302. doi: 10.1002/adma.202512302 (PMC12810622; doi:10.1002/adma.202512302)
Supplement: Supplementary file 1 — Supporting Information [file ADMA-38-e12302-s001.pdf]

ADVANCED MATERIALS

Supporting Information

for *Adv. Mater.*, DOI 10.1002/adma.202512302

Carrier Diffusion Links Single Crystal Quality and Photoluminescence in Halide Perovskite Radiation Detectors

*Zimu Wei, Khasim Saheb Bayikadi, Capucine Mamak, Milos Dubajic, Chieh-Szu Huang, Linfeng Pan, Mercouri G. Kanatzidis and Samuel D. Stranks**

Supporting Information

Carrier Diffusion Links Single Crystal Quality and Photoluminescence in Halide Perovskite Radiation Detectors

*Zimu Wei, Khasim Saheb Bayikadi, Capucine Mamak, Milos Dubajic, Chieh-Szu Huang, Linfeng Pan, Mercouri G. Kanatzidis, Samuel D. Stranks**

Zimu Wei, Capucine Mamak, Milos Dubajic, Chieh-Szu Huang, Linfeng Pan, Samuel D. Stranks
Department of Chemical Engineering and Biotechnology, University of Cambridge, Cambridge, CB3 0AS, UK
E-mail: sds65@cam.ac.uk

Khasim Saheb Bayikadi, Mercouri G. Kanatzidis
Department of Chemistry, Northwestern University, Evanston, Illinois 60208, USA

Experimental Section/Methods

Device fabrication and measurement: Two $CsPbBr_3$ crystals with different qualities were measured to compare detector performance across various γ-ray energies. A 1.5 mm-thick detector was fabricated with a 600 nm lead anode and a 150 nm gold guard ring (2 mm wide). The γ-ray response of this detector was measured at energies of 59.5 keV, 122 keV, and 662 keV from ^{241}Am, ^{57}Co, and ^{137}Cs, respectively, under an applied voltage of 400 V, with shaping times of 2 and 3 μs, and an electronic gain of 80. In addition, a second detector, 3.5 mm thick, was fabricated with the same electrode configuration (600 nm lead anode and 150 nm gold guard ring, 2 mm wide). Its γ-ray response was evaluated at energies of 122 keV, 662 keV, 1173 keV, and 1332 keV from ^{57}Co, ^{137}Cs, and ^{60}Co, under applied voltages of 700 V and 1000 V, with a shaping time of 6 μs and a gain of 80. Figure S2 provides clear evidence that the high-quality crystal consistently shows superior device performance, as seen from the 662 keV response, enabling operation at higher electric fields and resolution of hard γ-radiation above 1 MeV.

Materials characterisations: High-resolution SEM imaging was performed using a Gemini 800 system equipped with in-lens detectors. Measurements were conducted at an accelerating voltage of 5 kV with a 50-μm aperture.

Statistical Analysis: The full-spectrum fit was performed using the curve_fit routine from SciPy (version 1.12.0), which carries out nonlinear least-squares optimisation via the Trust Region Reflective algorithm. Parameter bounds were imposed to ensure physical plausibility of the fitted values. The free parameters were the first-order non-radiative recombination rate constant (k_1), the second-order radiative recombination coefficient (k_2), the diffusion coefficient (D), and the surface recombination velocity at the front surface (S_1). Uncertainties in the fitted parameters are reported as one standard error ($\pm 1\sigma$), calculated from the square roots of the diagonal elements of the covariance matrix returned by the fitting routine. Sample sizes (n) for each experiment are specified in the figure captions and/or the main text. All PL analyses were performed using Python.

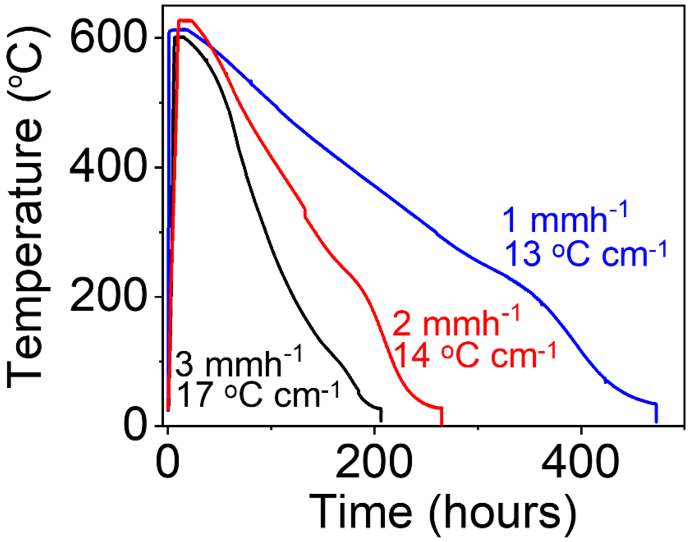

Figure S1. Growth profiles of $CsPbBr_3$ single crystals obtained under controlled temperature gradient and different growth rates.

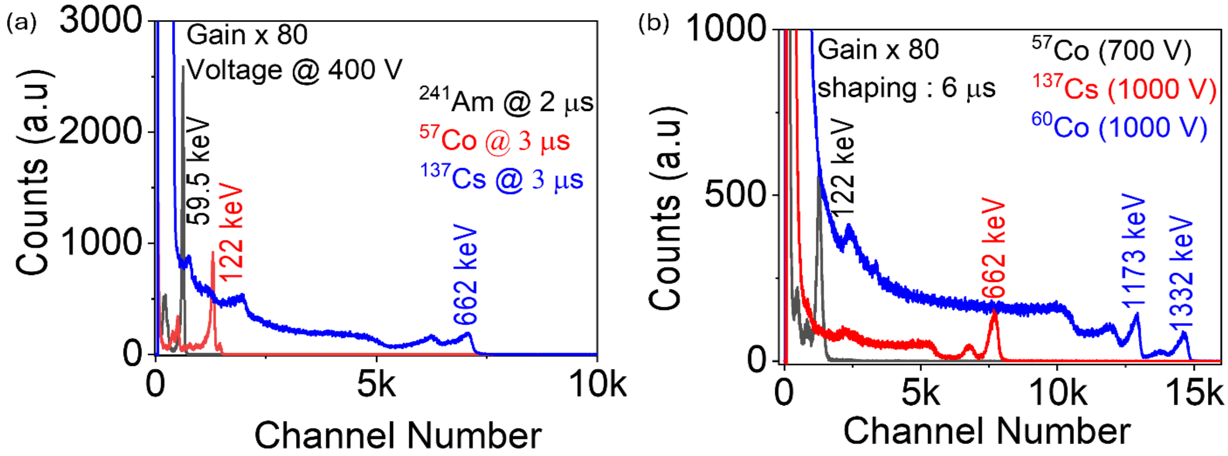

Figure S2. (a) γ-ray response of a 1.5 mm-thick CsPbBr3 detector using a low-quality crystal, measured at an applied voltage of 400 V and an electronic gain of 80, using shaping times of 2 and 3 μs for different sources: ^{241}Am, ^{57}Co, and ^{137}Cs. (b) γ-ray response of a 3.5 mm-thick $CsPbBr_3$ detector with a high-quality crystal, measured at applied voltages of 700 V and 1000 V and an electronic gain of 80, using a shaping time of 6 μs for different sources: ^{57}Co, ^{137}Cs, and ^{60}Co.

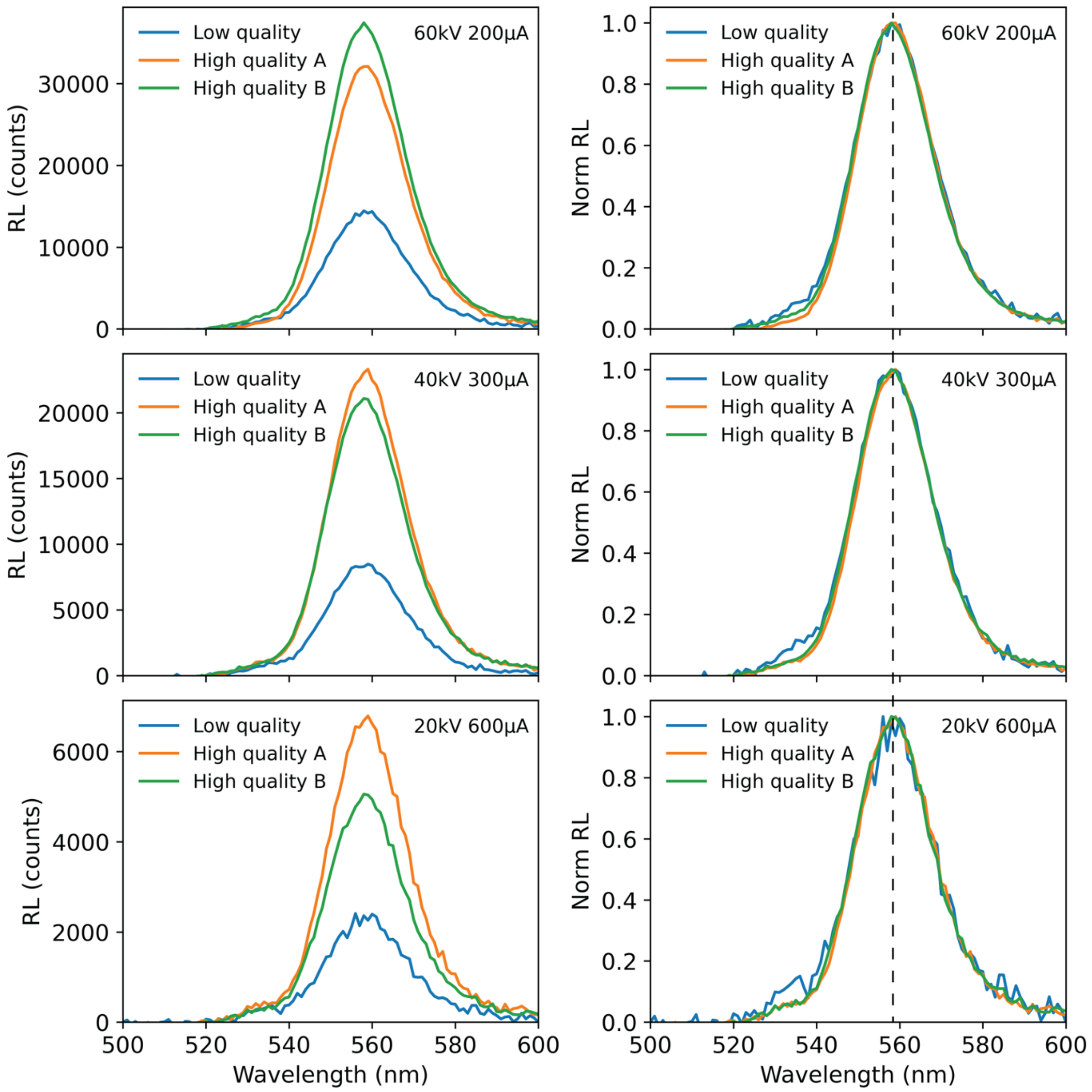

Figure S3. Radioluminescence (RL) spectra of $CsPbBr_3$ single crystals measured at varying incident radiation energy (60kV, 40kV and 20kV). All RL spectra were collected in a transmission configuration (i.e. excitation and detection from opposite sides).

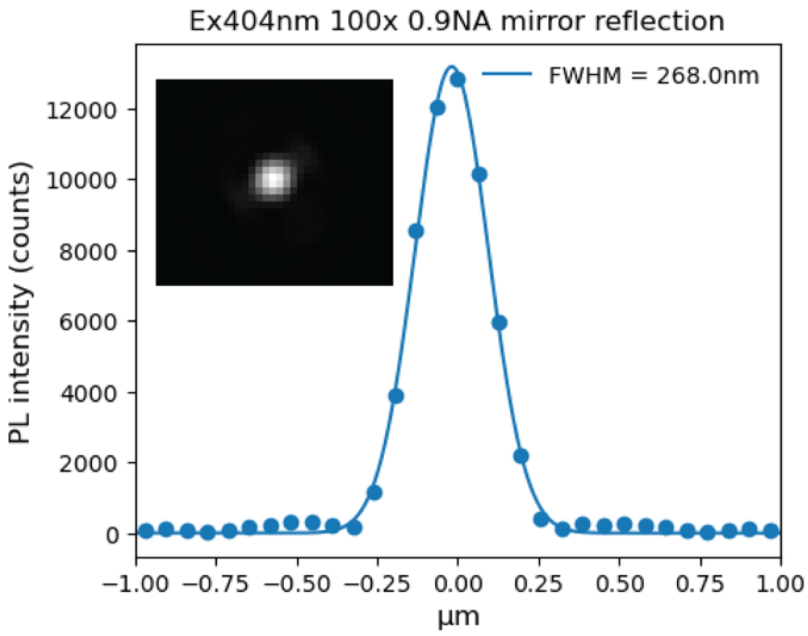

Figure S4. The focused laser beam at a wavelength of 404 nm in the confocal microscope, measured via mirror reflection. The full width at half maximum (FWHM) of the spot size is found to be 270 nm using a Gaussian fit. The inset shows the 2D image of the laser beam.

Supplementary Note 1. Estimation of axial resolution in confocal microscopy

The axial full width half maximum (FWHM) of the detection point spread function (PSF) is estimated by

$$\mathrm{FWHM}_{\mathrm{axial,\ pinhole}\sim 1\mathrm{AU}} = \frac{0.88\,\lambda_0}{n-\sqrt{n^2-\mathrm{NA}^2}}.$$

Here, n is the refractive index of the medium ($n_{\mathrm{air}} = 1$), and λ_0 is calculated as an average wavelength according to

$$\lambda_0 = \sqrt{2}\frac{\lambda_{\mathrm{em}}\lambda_{\mathrm{ex}}}{\sqrt{\lambda_{\mathrm{ex}}^2+\lambda_{\mathrm{em}}^2}}.$$

We use 404 nm and 530 nm as the excitation and emission wavelengths, respectively.

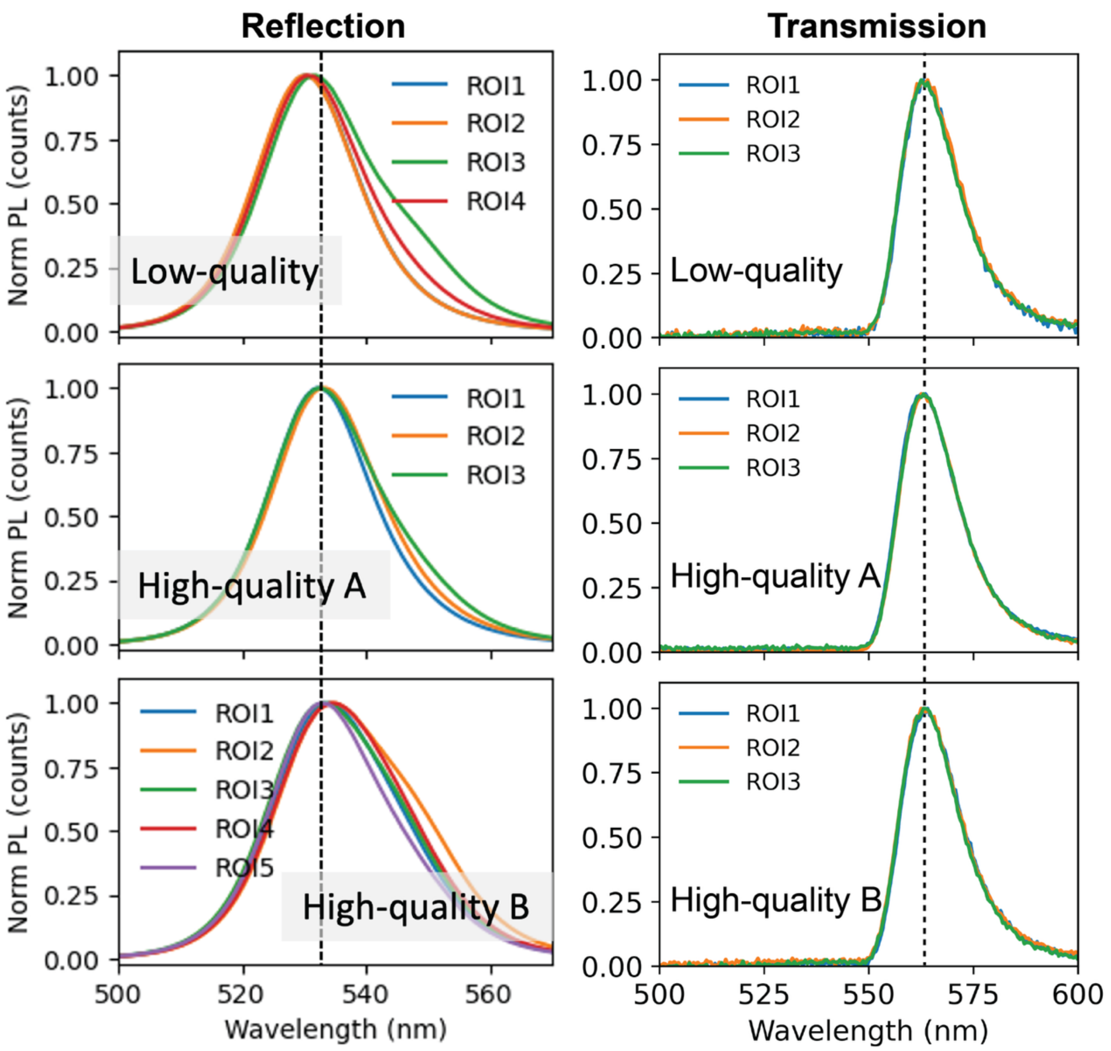

Figure S5. Macroscopic PL spectra of $CsPbBr_3$ single crystals across different regions of interest (ROIs) measured in the reflection (left panel) and transmission (right panel) configurations. Despite ROI-dependent shoulder behaviour, the PL peak position shows a consistent redshift in high-quality crystals in the reflection mode, while transmitted PL are the same across different crystals due to the complete self-reabsorption.

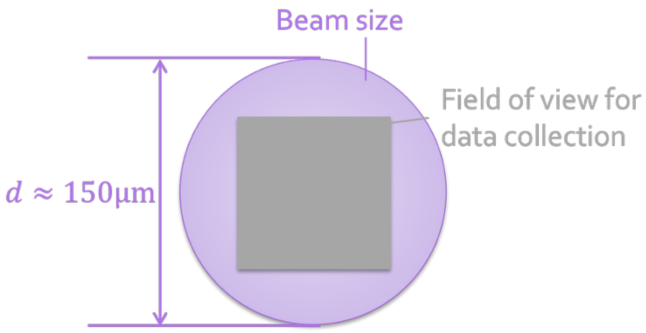

Figure S6. Schematic top view of time-resolved PL spectra collection with a widefield microscope.

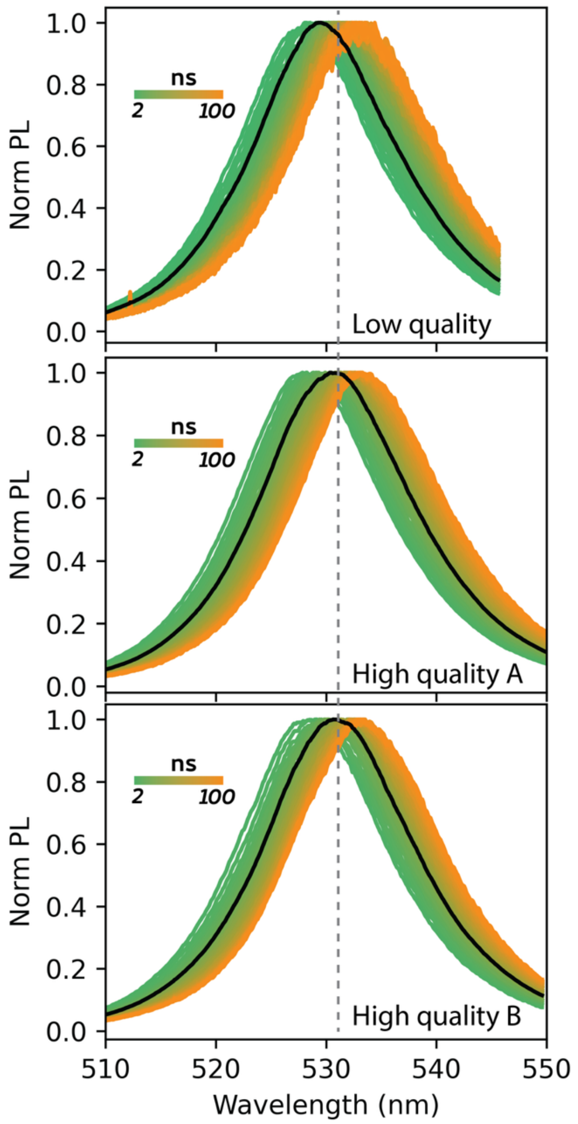

Figure S7. Normalised PL spectra over 2–100 ns for $CsPbBr_3$ crystals of varying quality. The integrated spectra are shown as black curves, calculated by summing the 2–100 ns PL spectra based on the original intensity and then normalised for peak position comparison. The dashed line serves as a visual guide for comparison.

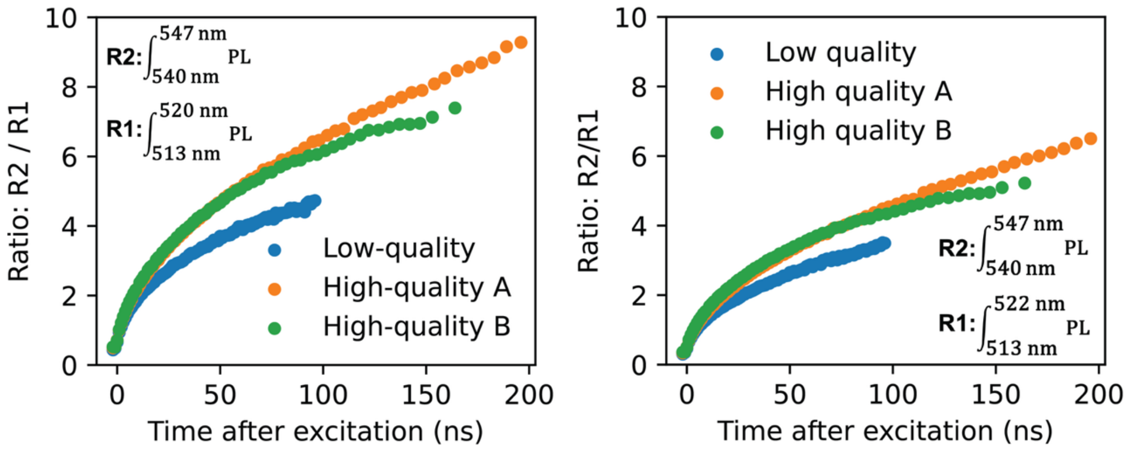

Figure S8. Experimental ratio (R2/R1) as a function of time across different selected ranges.

Supplementary Note 2. Consideration of the photon-recycling effect on measured PL

Photon recycling is known to enhance the optoelectronic properties of thin films[1,2]. However, its impact on the measured PL of single crystals is limited for several reasons. Given the millimetre-scale thickness and the reported photon-recycling effective length (1–4 µm)[3,4], only a small fraction of the isotropic emission following each recycling event can reach the crystal surface and be detected. Photons emitted at large angles either exit through the opposite side of the crystal or undergo internal reflection and eventually out-couple from the same side, but at locations far from the excitation area (in thin films, such contributions are typically observed tens of micrometres away). Since our measurements are collected within the excitation area in reflection mode using a widefield microscope, neither scenario greatly contributes to the detected PL. This reasoning is supported by several reports, which show photon recycling-induced external emission as low as 0.5% in perovskite single crystals[5], as well as simulations indicating that photon recycling only becomes significant at long times and large depths in transmission-mode measurements[4].

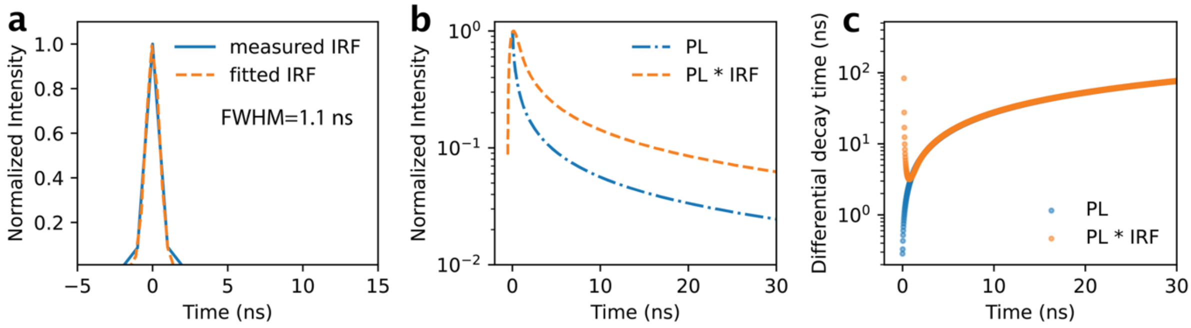

Figure S9. The convolution effect with the instrument response function (IRF) on simulated TRPL decay and differential decay time. (a) The IRF of the electron-multiplied intensified CCD camera at a 1 ns gate width, measured via mirror reflection. The full width at half maximum (FWHM) of the IRF is found to be 1.1 ns using a Gaussian fit. The Gaussian fit was then used as the IRF for convolution in the numerical simulations. (b) Numerical simulation shows that the IRF convolution results in an apparently slower TRPL decay with normalisation. (c) Converting the TRPL decay into differential decay times minimises the effect of IRF convolution on the long time scales.

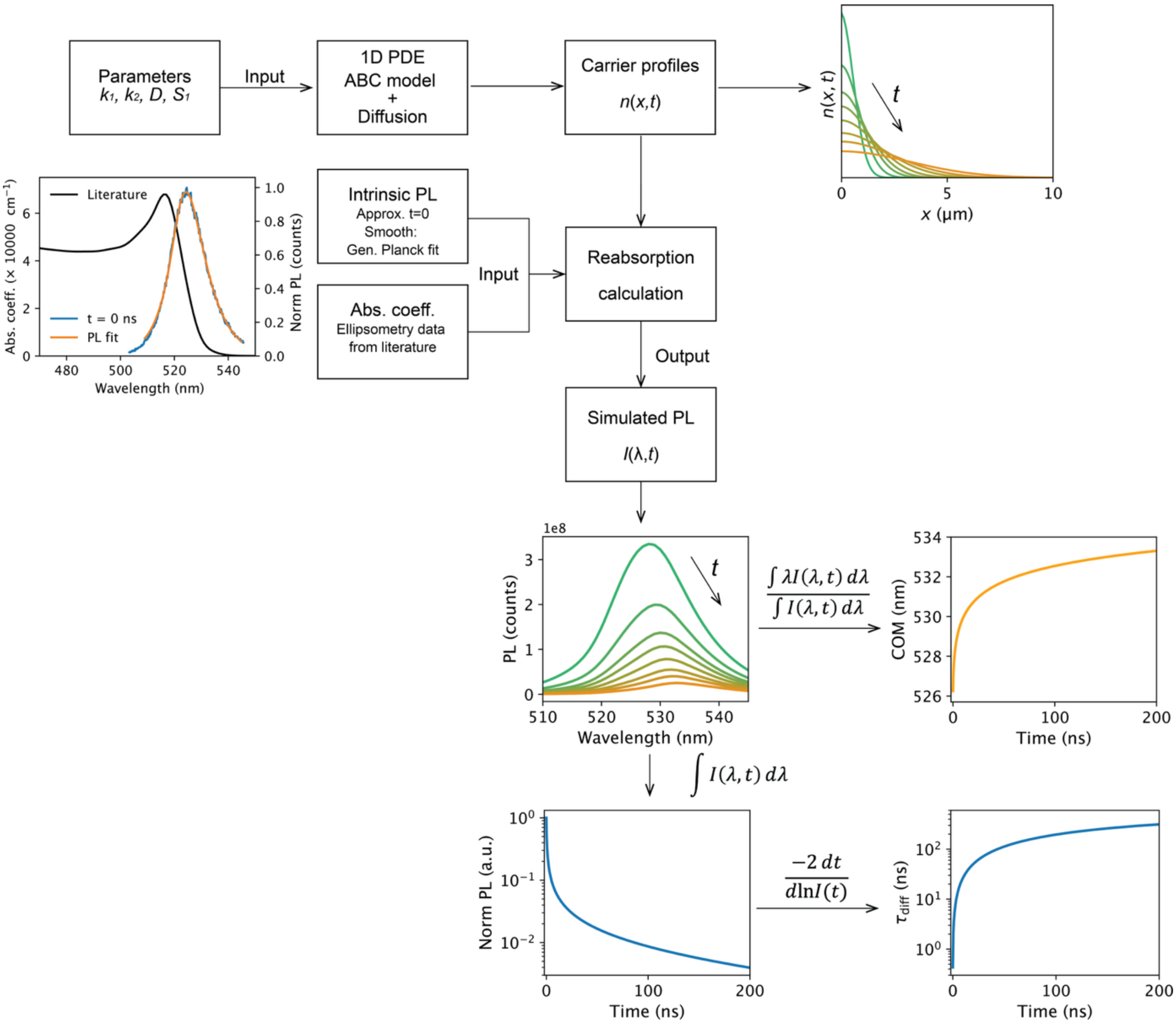

Figure S10. Flowchart of simulating time-resolved PL spectra using the numerical model described in the main text.

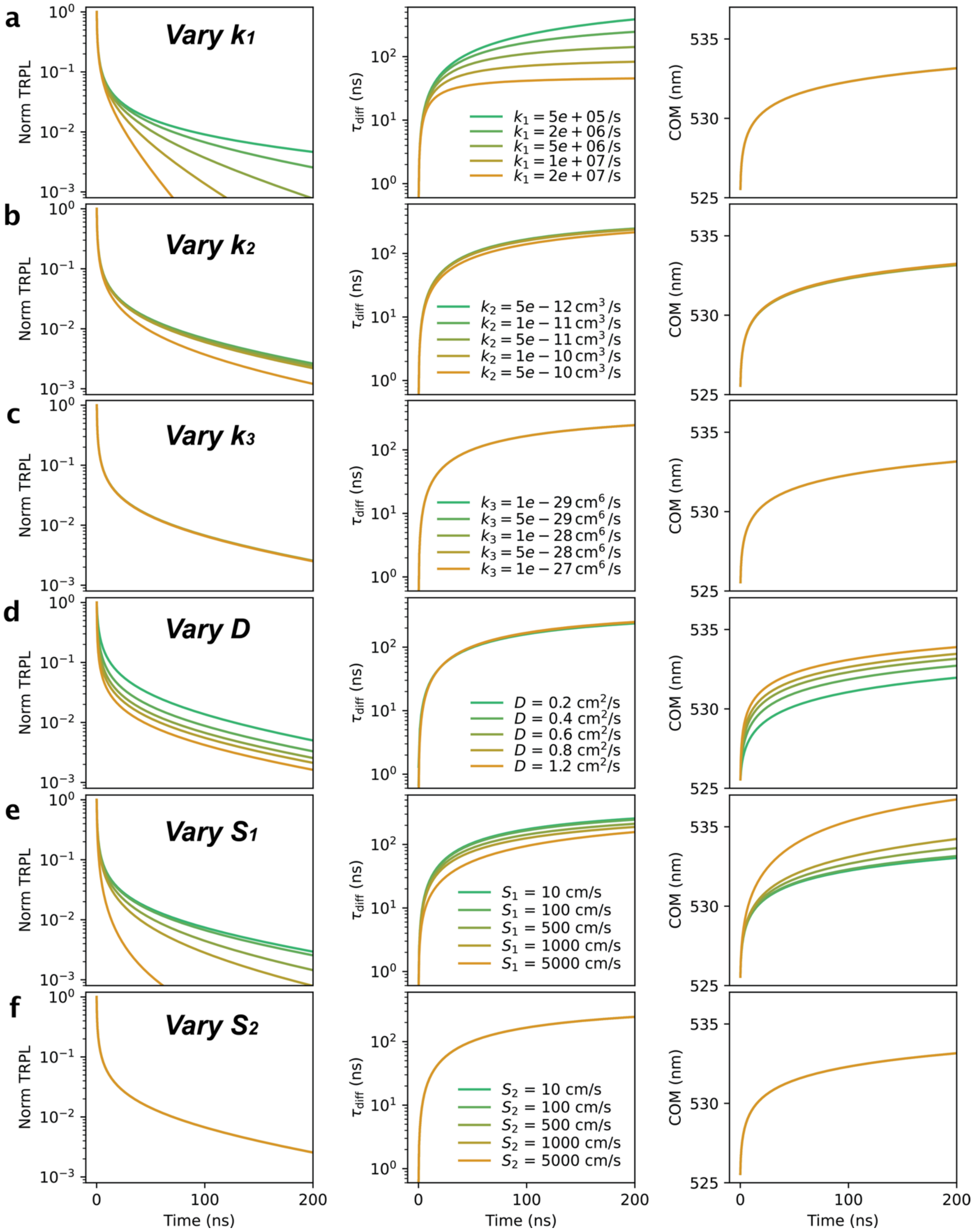

Figure S11. Effect of varying simulation parameters on the PL dynamics, differential decay times (τ_{diff}), and centre of mass (COM) as a function of time: (a) varying trap-assisted recombination rate k_1, (b) varying radiative recombination rate k_2, (c) varying Auger recombination rate k_3, (d) varying diffusion coefficient D, (e) varying front surface recombination velocity S_1 and (f) varying back surface recombination velocity S_2. Default parameters: initial carrier density $n_0 = 1.4 \times 10^{17}\mathrm{cm}^{-3}$, $k_1 = 2 \times 10^6 \mathrm{s}^{-1}$, $k_2 = 2 \times 10^{-11} \mathrm{cm}^3\mathrm{s}^{-1}$, $k_3 = 1 \times 10^{-28} \mathrm{cm}^6\mathrm{s}^{-1}$, $D = 0.6\ \mathrm{cm}^2\mathrm{s}^{-1}$, $S_1 = 100\ \mathrm{cm\ s}^{-1}$ and $S_2 = 100\ \mathrm{cm\ s}^{-1}$.

Supplementary Note 3. Validation of the full-spectrum fit

To extract different parameters, common approaches rely on manually comparing the simulation with condensed PL observables, such as differential decay times and centre of mass, to find parameters that can better match these observables. Here, we performed a full-spectrum fit by wrapping the PL spectra at each time step into the model for curve_fit. To validate the full-spectrum fitting method, we first applied it to synthetic data with known parameter values. As shown in Figure S12, fitting without the full spectra produces a broader distribution of fitted values and is more strongly influenced by initial conditions. In contrast, the full-spectrum fit accurately recovers the true parameters regardless of the initial guesses.

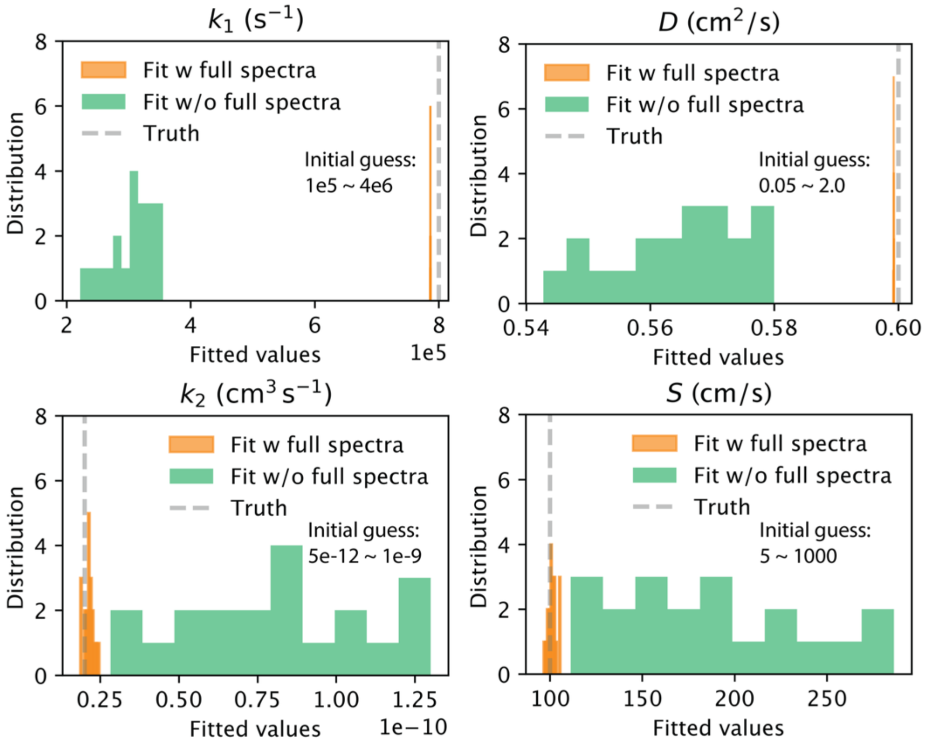

Figure S12. Curve fitting of synthetic data with varied initial guesses, comparing results obtained with and without the full time-resolved spectra.

Table S1. List of input and fitted parameters

	Low-quality A	Low-quality B	High-quality A	High-quality B	High-quality C
**Input parameters:**					
Initial charge carrier density, measured $n_0 (\times 10^{17} \mathrm{cm}^{-3})$	1.56	1.36	1.56	1.56	1.36
Absorption coefficient at 398 nm, literature[6] $\alpha_{\mathrm{ex}} (\times 10^{4} \mathrm{cm}^{-1})$	8.2	8.2	8.2	8.2	8.2
Auger recombination rate* $k_3 (\mathrm{cm}^{6} \mathrm{s}^{-1})$	0	0	0	0	0
Back surface recombination velocity* $S_2 (\mathrm{cm\ s}^{-1})$	0	0	0	0	0
**Fitted parameters:**					
Trap-assisted recombination rate $k_1 (\times 10^{6} \mathrm{s}^{-1})$	16.2 ± 3.3	17.8 ± 4.2	1.2 ± 0.3	4.4 ± 2.0	1.1 ± 0.3
Radiative recombination rate $k_2 (\times 10^{-11} \mathrm{cm}^{3} \mathrm{s}^{-1})$	8.8 ± 22	6.8 ± 25	6.1 ± 28	7.7 ± 27	6.5 ± 19
Diffusion coefficient $D (\mathrm{cm}^{2} \mathrm{s}^{-1})$	0.51 ± 0.03	0.41 ± 0.02	0.65 ± 0.02	0.59 ± 0.04	0.60 ± 0.02
Front surface recombination velocity $S_1 (\mathrm{cm\ s}^{-1})$	52 ± 240	41 ± 320	8.4 ± 440	58 ± 400	58 ± 290

Note: Parameters marked with an asterisk are set as zero because they have minimal impact on the simulation due to the initial carrier density and crystal thickness.

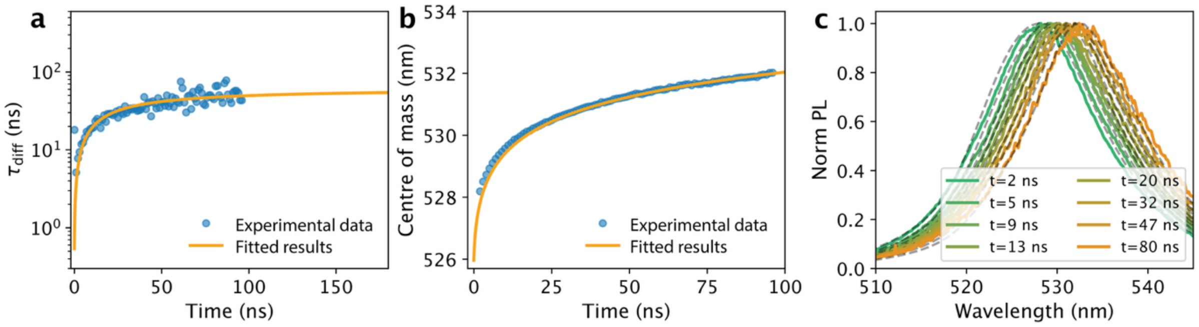

Figure S13. Fitted results for the low-quality crystal A using the full-spectrum fitting procedure. (a) Differential decay times ($\tau_{\rm diff}$) and the fit. (b) Centre of mass as a function of time and the fit. (c) Time-resolved PL spectra and their fits. All fitted parameters are listed in Table S1.

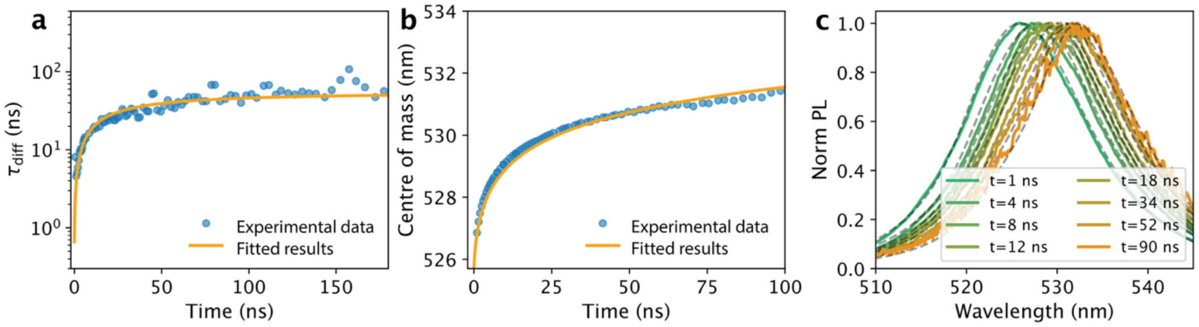

Figure S14. Fitted results for the low-quality crystal B using the full-spectrum fitting procedure. (a) Differential decay times ($\tau_{\rm diff}$) and the fit. (b) Centre of mass as a function of time and the fit. (c) Time-resolved PL spectra and their fits. All fitted parameters are listed in Table S1.

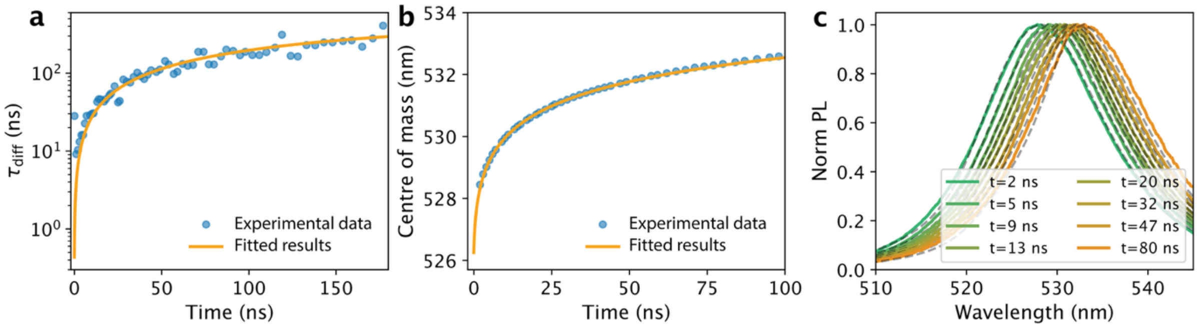

Figure S15. Fitted results for the high-quality crystal A using the full-spectrum fitting procedure. (a) Differential decay times ($\tau_{\rm diff}$) and the fit. (b) Centre of mass as a function of time and the fit. (c) Time-resolved PL spectra and their fits. All fitted parameters are listed in Table S1.

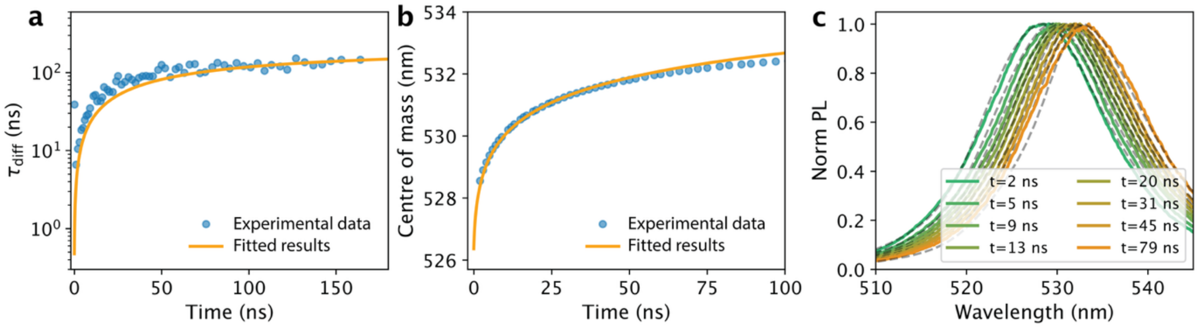

Figure S16. Fitted results for the high-quality crystal B using the full-spectrum fitting procedure. (a) Differential decay times (τ_{diff}) and the fit. (b) Centre of mass as a function of time and the fit. (c) Time-resolved PL spectra and their fits. All fitted parameters are listed in Table S1.

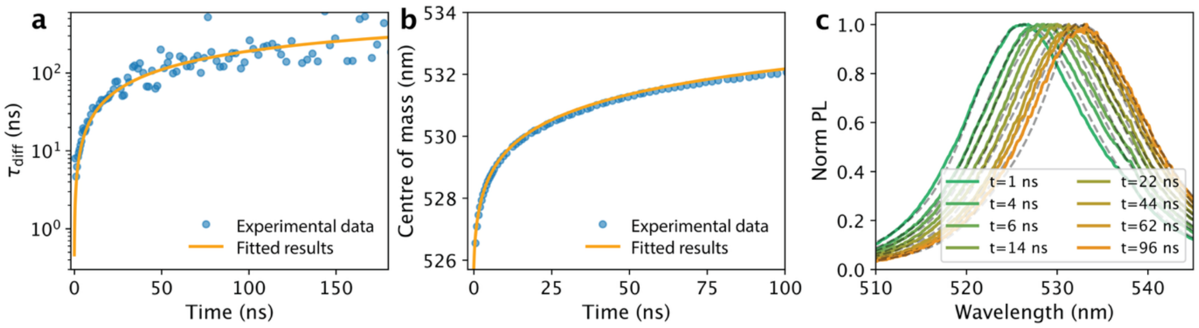

Figure S17. Fitted results for the high-quality crystal C using the full-spectrum fitting procedure. (a) Differential decay times (τ_{diff}) and the fit. (b) Centre of mass as a function of time and the fit. (c) Time-resolved PL spectra and their fits. All fitted parameters are listed in Table S1.

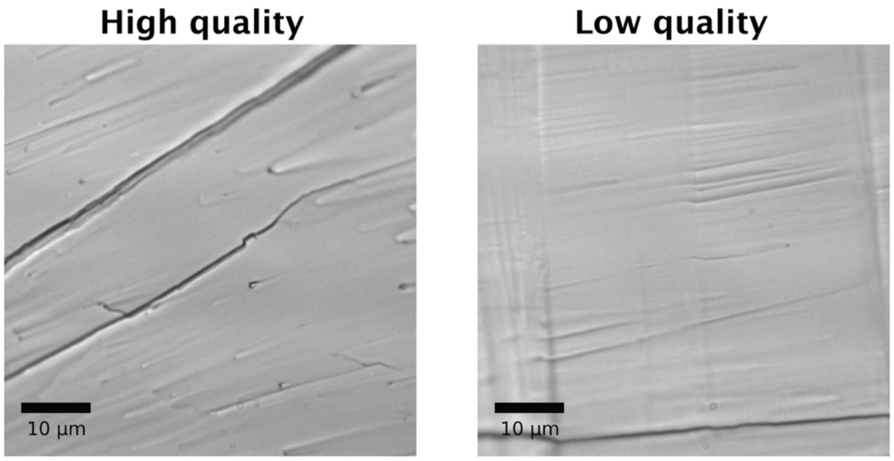

Figure S18. White-light reflection images of $CsPbBr_3$ single crystals showing the same region as in Figures 4a and 4b.

Supplementary Note 4. Depth of focus in two-photon excitation microscopy.

Due to the refractive index mismatch between air (n_1=1) and perovskite single crystals (n_2 ≈2.2)[7], refraction causes the light to focus deeper into the crystal. As illustrated in Figure S13, the actual offset (ΔZ) resulting from this aberration increases with the incident angle (ΔZ), meaning that an overfilled high-numerical-aperture objective with a large maximum incidence angle (θ_{max}) will experience stronger aberration effects. Additionally, the offset ΔZ scales linearly with the nominal focus position (Z), causing the depth of focus to transition from being diffraction-limited (~2 μm) to aberration-limited as the focal plane moves deeper into the crystal. The depth of focus due to aberration can be estimated using the following expression[8]

$$\text{d.o.f.}=Z\left[\left[\frac{\text{NA}^2\left(\left(\frac{n_2}{n_1}\right)^2-1\right)}{(1-\text{NA}^2)}+\left(\frac{n_2}{n_1}\right)^2\right]^{1/2}-\frac{n_2}{n_1}\right].$$

Applying this estimation yields depths of focus of 4.5 μm and 6.8 μm at Z=-4 μm and Z=-6 μm, respectively. Thus, both the actual focal position and the depth of focus increase as the laser is focused deeper into the crystal, resulting in a lower excitation density at larger depths.

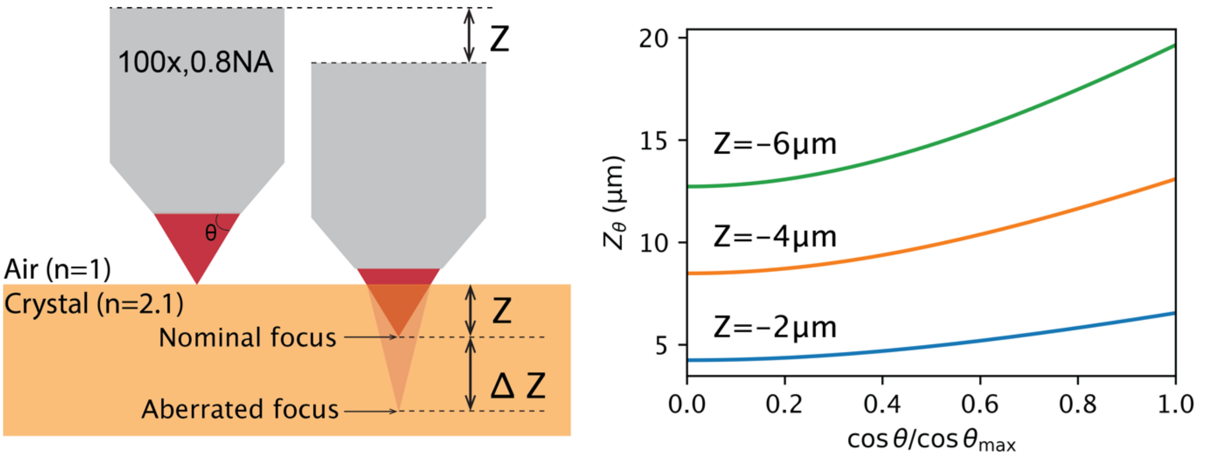

Figure S19. Aberration effects and depth of focus in two-photon excitation microscopy. Left: Schematic of a 100×, 0.8 NA objective focusing through air (n = 1) into a perovskite single crystal (n ≈ 2.2), illustrating the offset (ΔZ) between nominal focus (Z) and actual focus due to aberration. θ denotes the light incidence angle. Right: Distribution of actual focal positions across incident angles for a given nominal focus (Z).

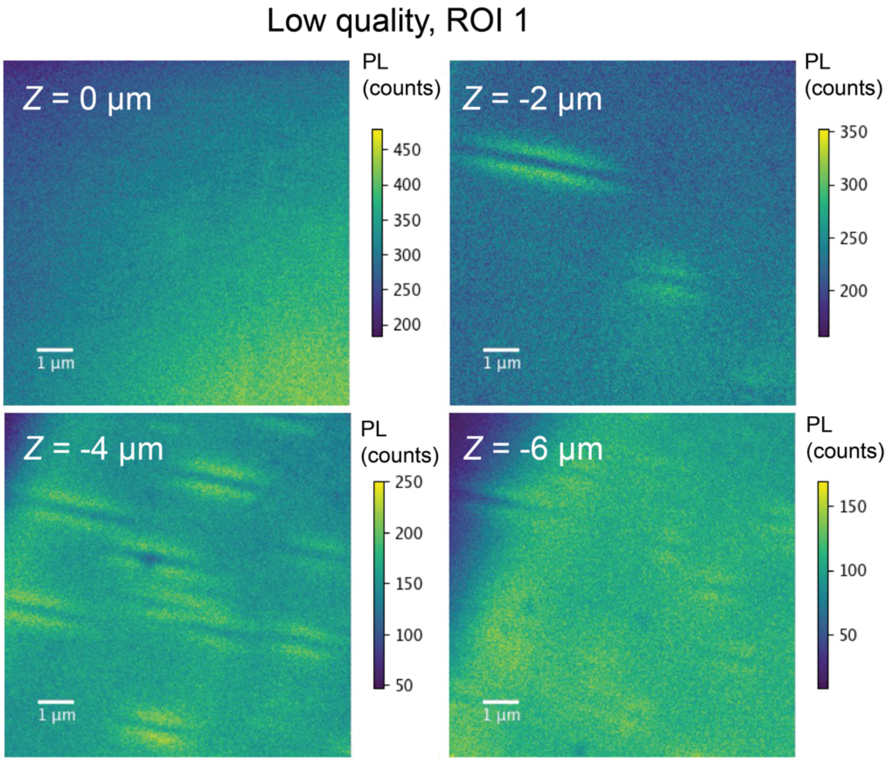

Figure S20. 2P PL intensity map of the low-quality crystal at different depths. The region of interest is the same as in the main text.

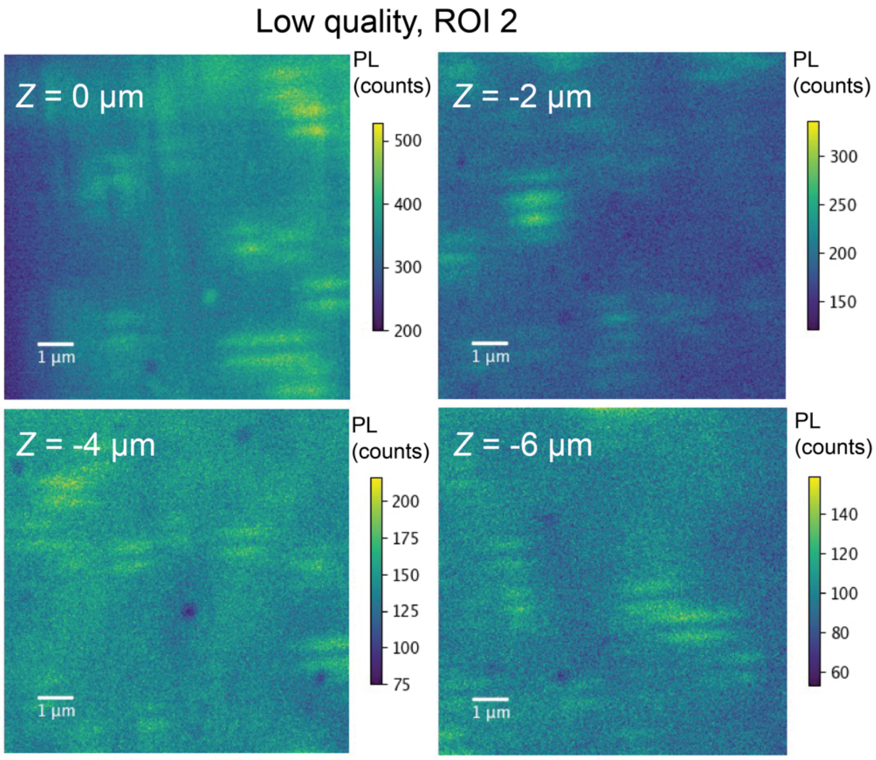

Figure S21. 2P PL intensity map of the low-quality crystal at different depths. The region of interest is different from that in the main text.

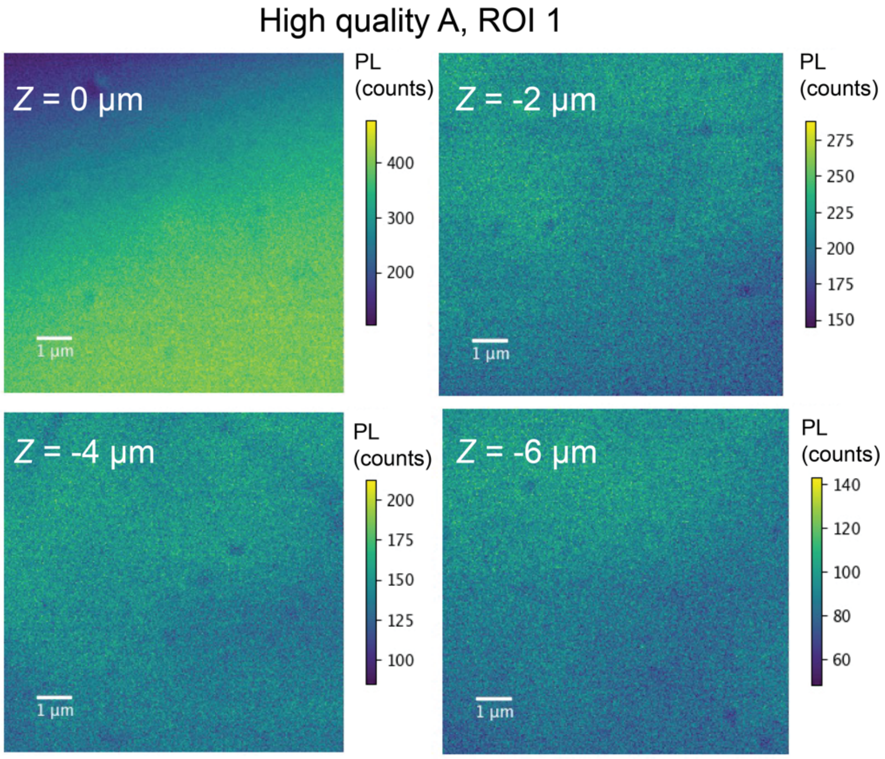

Figure S22. 2P PL intensity map of the high-quality crystal A at different depths. The region of interest is the same as in the main text.

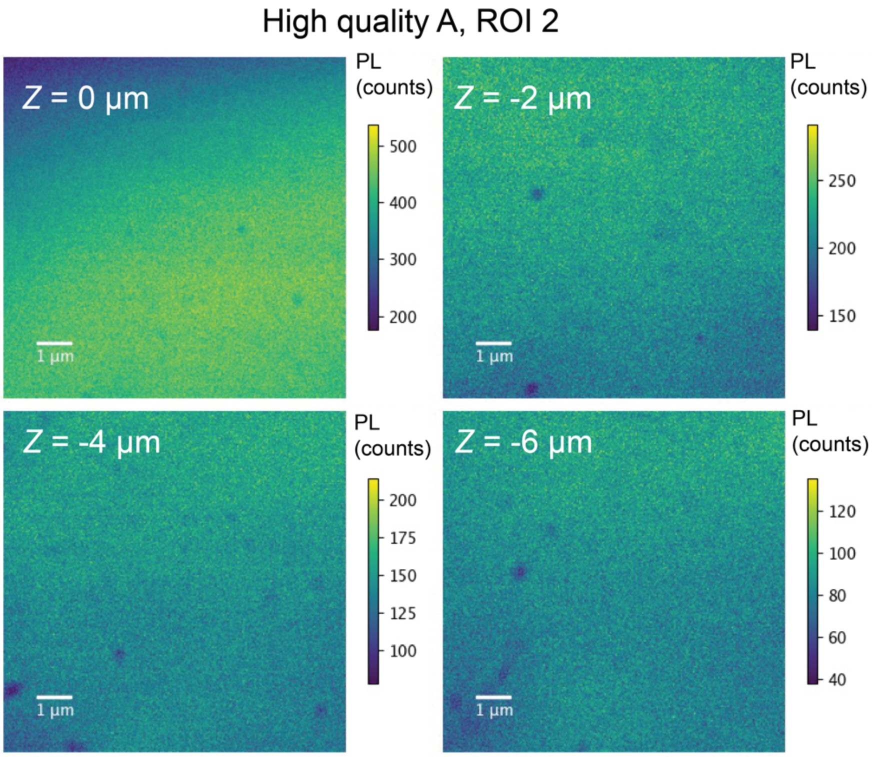

Figure S23. 2P PL intensity map of the high-quality crystal A at different depths. The region of interest is different from that in the main text.

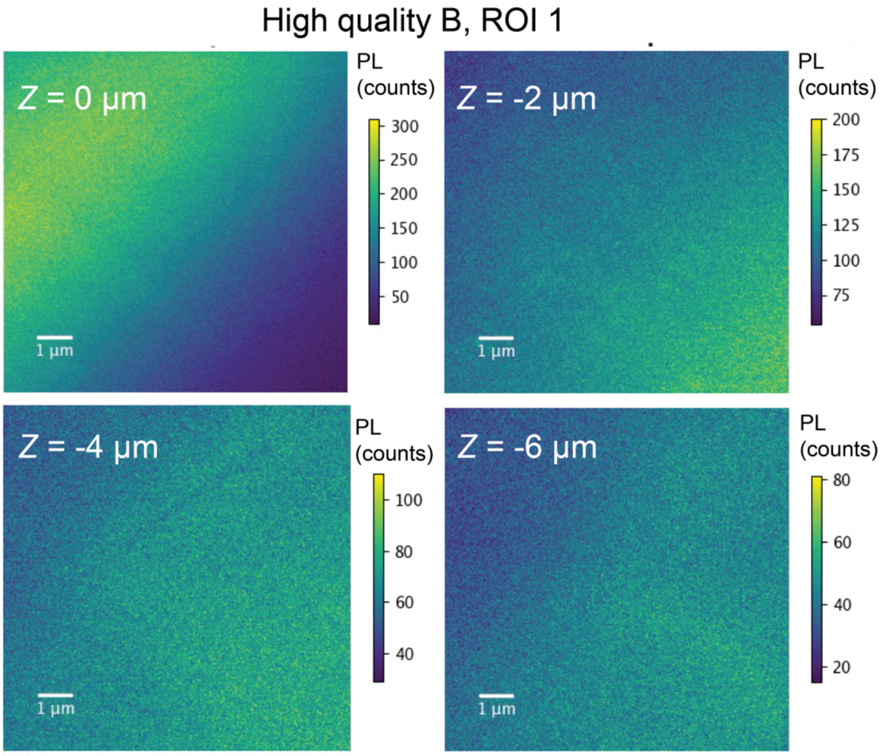

Figure S24. 2P PL intensity map of the high-quality crystal B at different depths.

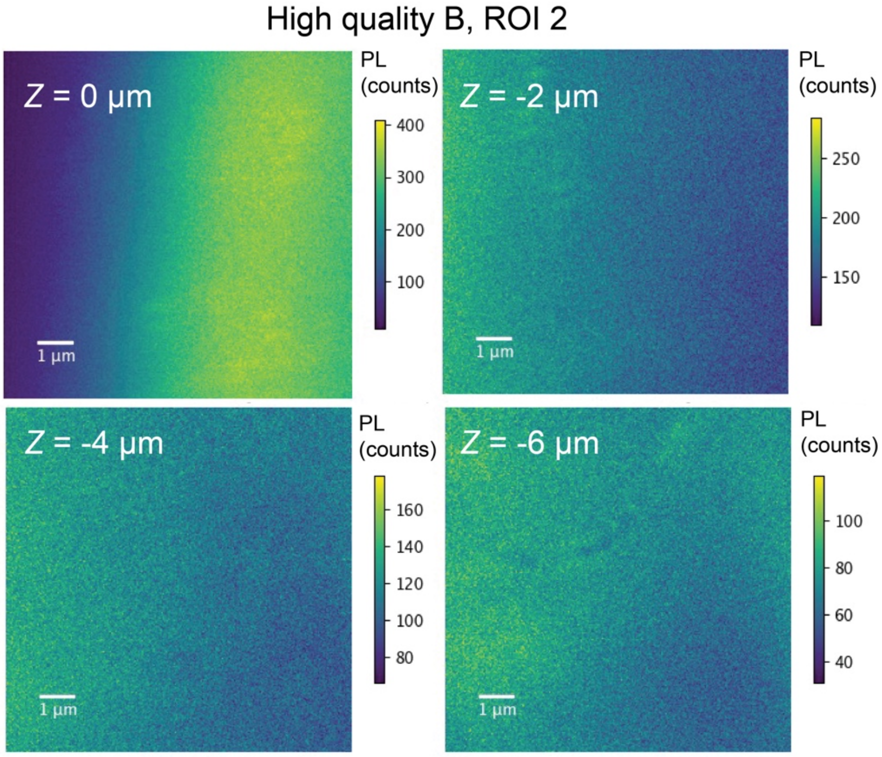

Figure S25. 2P PL intensity map of the high-quality crystal B at different depths. The region of interest is different from that in Figure S24.

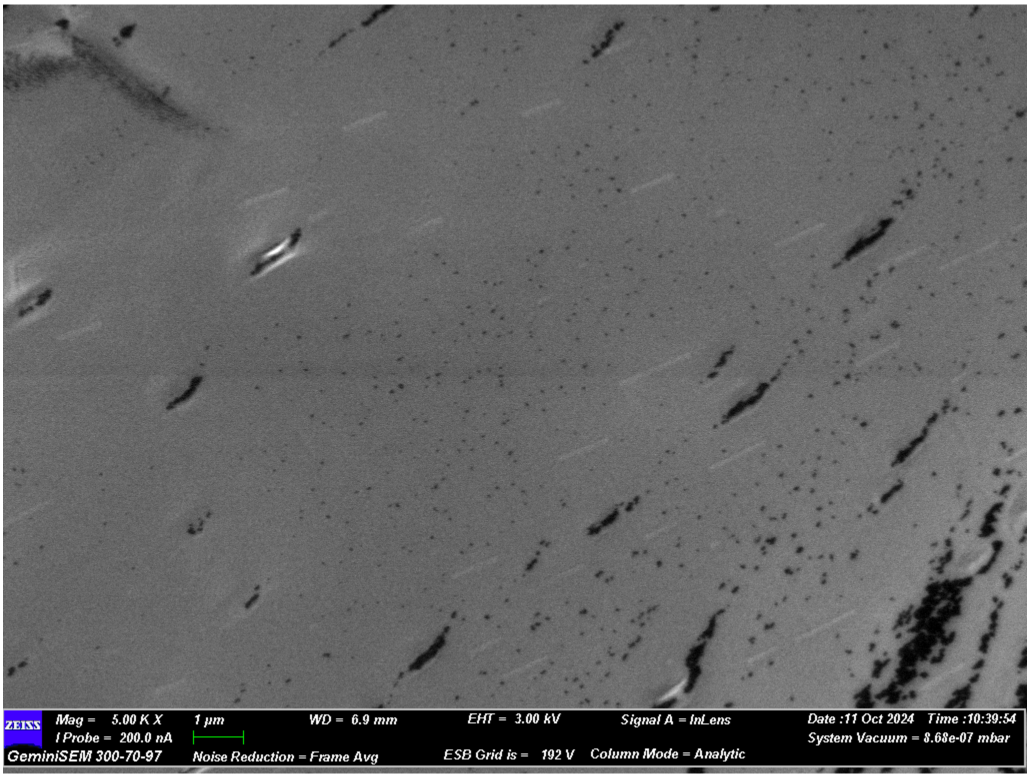

Figure S26. Scanning electron micrograph of a cleaved low-quality crystal showing microscale stripes consistent with PL maps.

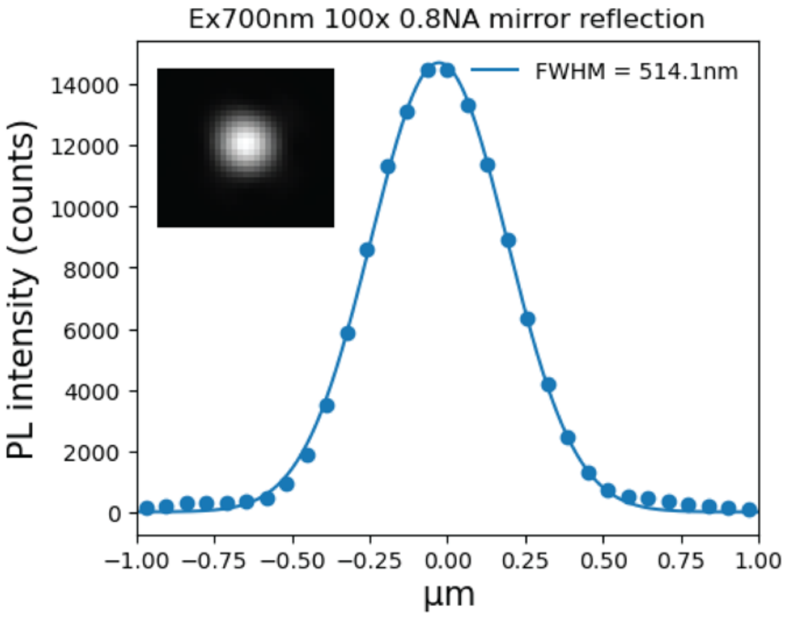

Figure S27. The focused laser beam at a wavelength of 700 nm in the confocal microscope, measured via mirror reflection. The full width at half maximum (FWHM) of the spot size is found to be 514 nm using a Gaussian fit. The inset shows the 2D image of the laser beam.

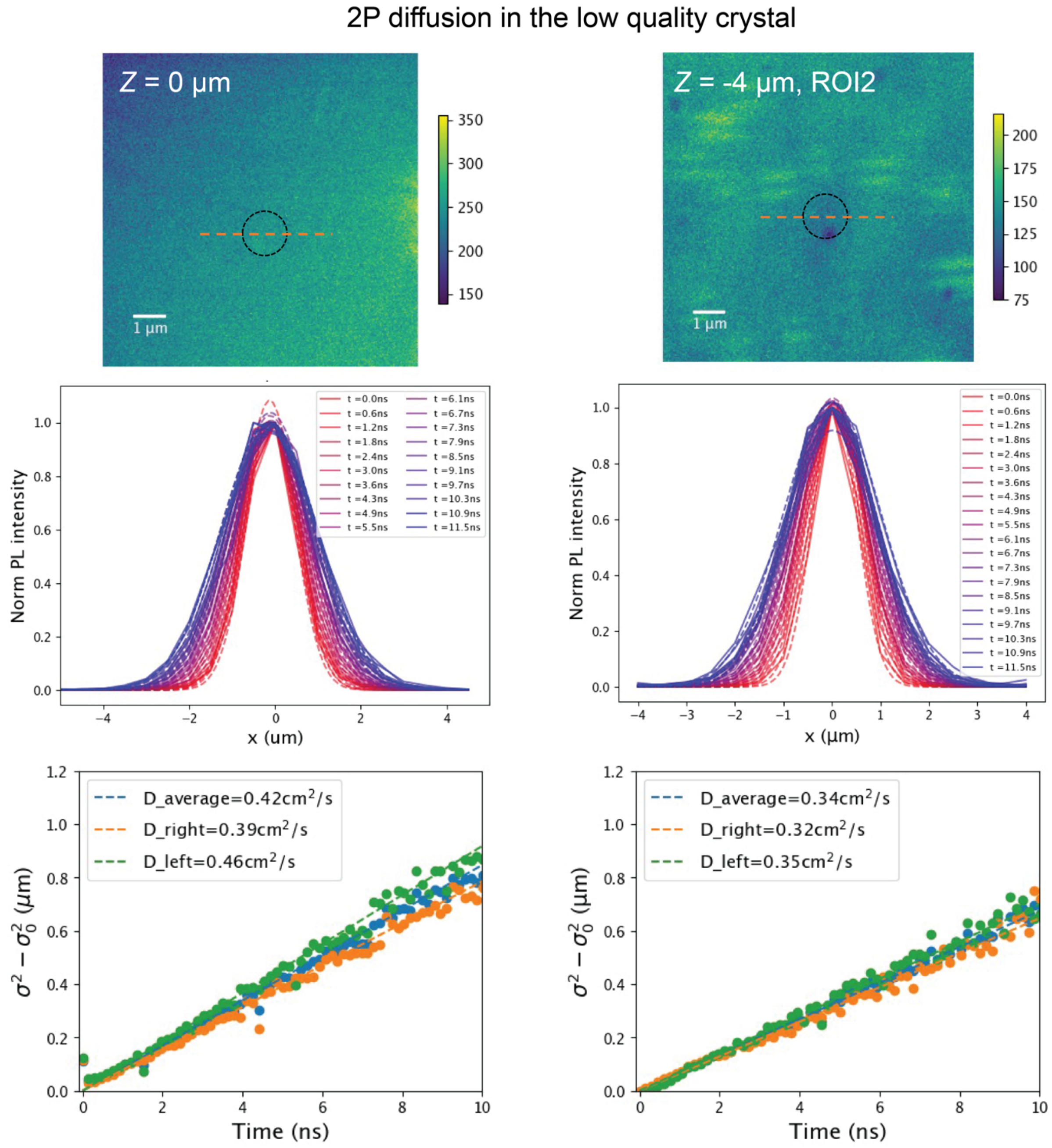

Figure S28. 2P diffusion results in the low-quality crystal at Z = 0 µm (left panel) and Z = -4 µm (right panel), from a different ROI than that shown in the main text. Both diffusion profiles are symmetrical when the line scan is performed away from defects.

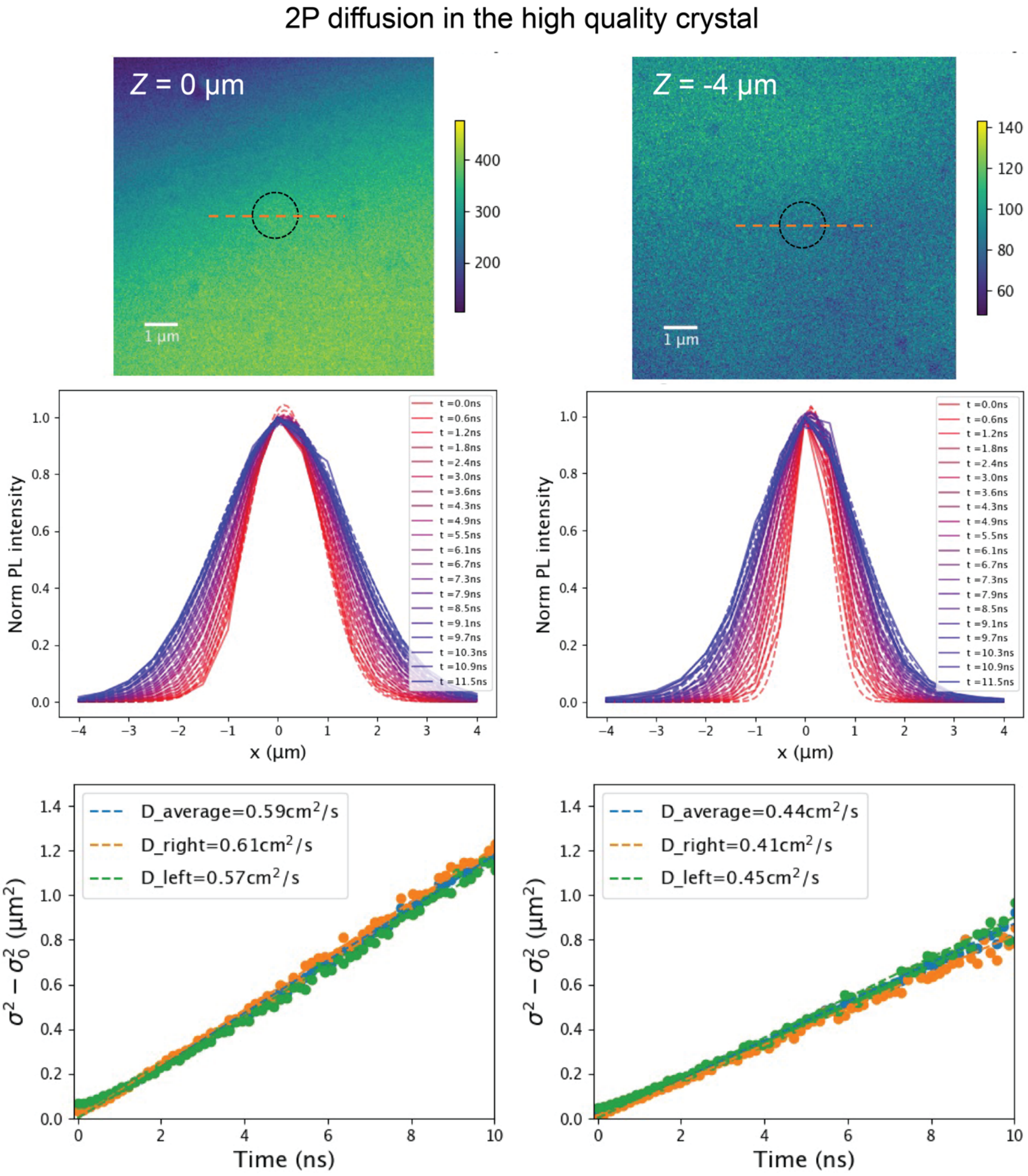

Figure S29. 2P diffusion results in the high-quality crystal at *Z* = 0 µm (left panel) and *Z* = -6 µm (right panel). Both diffusion profiles are symmetrical when the line scan is performed in the absence of defects.

Reference

[1] L. M. Pazos-Outón, M. Szumilo, R. Lamboll, J. M. Richter, M. Crespo-Quesada, M. Abdi-Jalebi, H. J. Beeson, M. Vrućinić, M. Alsari, H. J. Snaith, B. Ehrler, R. H. Friend, F. Deschler, *Science* **2016**, *351*, 1430.

[2] C. Cho, B. Zhao, G. D. Tainter, J.-Y. Lee, R. H. Friend, D. Di, F. Deschler, N. C. Greenham, *Nat Commun* **2020**, *11*, 611.

[3] T. Yamada, Y. Yamada, Y. Nakaike, A. Wakamiya, Y. Kanemitsu, *Phys. Rev. Appl.* **2017**, *7*, 014001.

[4] F. Staub, I. Anusca, D. C. Lupascu, U. Rau, T. Kirchartz, *J. Phys. Mater.* **2020**, *3*, 025003.

[5] Y. Fang, H. Wei, Q. Dong, J. Huang, *Nat Commun* **2017**, *8*, 14417.

[6] M. C. Brennan, D. M. Krein, E. Rowe, C. L. McCleese, L. Sun, K. G. Berry, P. R. Stevenson, M. A. Susner, T. A. Grusenmeyer, *MRS Communications* **2024**, *14*, 900.

[7] G. Ermolaev, A. P. Pushkarev, A. Zhizhchenko, A. A. Kuchmizhak, I. Iorsh, I. Kruglov, A. Mazitov, A. Ishteev, K. Konstantinova, D. Saranin, A. Slavich, D. Stosic, E. S. Zhukova, G. Tselikov, A. Di Carlo, A. Arsenin, K. S. Novoselov, S. V. Makarov, V. S. Volkov, *Nano Lett.* **2023**, *23*, 2570.

[8] N. J. Everall, *Appl. Spectrosc., AS* **2000**, *54*, 773.
